# Hepatic Form of Dihydrolipoamide Dehydrogenase Deficiency (DLDD): Phenotypic Spectrum, Laboratory Findings, and Therapeutic Approaches in 52 Patients

**DOI:** 10.1002/jimd.70035

**Published:** 2025-05-19

**Authors:** Nicole Hammann, Christian Staufner, Lea Dewi Schlieben, Antal Dezsőfi‐Gottl, René G. Feichtinger, Johannes Häberle, Norman Junge, Vassiliki Konstantopoulou, Robert Kopajtich, Valérie McLin, Daisy Rymen, Christoph Slavetinsky, Ekkehard Sturm, Johannes A. Mayr, Matias Wagner, Stefan Kölker, Holger Prokisch, Georg F. Hoffmann, Dominic Lenz

**Affiliations:** ^1^ Heidelberg University, Medical Faculty, University Hospital Heidelberg, Center for Child and Adolescent Medicine Division of Pediatric Neurology and Metabolic Medicine Heidelberg Germany; ^2^ Institute of Human Genetics, School of Medicine and Health Technical University of Munich Munich Germany; ^3^ Institute of Human Genetics Helmholtz Zentrum Munich Neuherberg Germany; ^4^ Bókay Street Department, Pediatric Centre Semmelweis University Budapest Hungary; ^5^ University Children's Hospital Salzburger Landeskliniken (SALK) and Paracelsus Medical University (PMU) Salzburg Austria; ^6^ University Children's Hospital Zurich and Children's Research Center Zurich Switzerland; ^7^ Division for Paediatric Gastroenterology and Hepatology, Department of Paediatric Kidney, Liver and Metabolic Diseases Hannover Medical School Hannover Germany; ^8^ Department of Pediatrics and Adolescent Medicine Medical University of Vienna Vienna Austria; ^9^ Swiss Pediatric Liver Center Geneva University Hospitals Geneva Geneva Switzerland; ^10^ Center for Metabolic Diseases, Department of Paediatrics University Hospitals Leuven Leuven Belgium; ^11^ Paediatric Surgery and Urology University Children's Hospital Tübingen Tübingen Germany; ^12^ Paediatric Gastroenterology and Hepatology University Children's Hospital Tübingen Tübingen Germany; ^13^ Department Computational Health, Institute of Neurogenomics Helmholtz Zentrum München Munich Germany

**Keywords:** DLD, E3, hepatic mitochondriopathy, mitochondrial disease, pediatric acute liver failure

## Abstract

Dihydrolipoamide dehydrogenase deficiency (MIM 246900/DLDD) is an autosomal recessive mitochondrial disease with three clinical subgroups. The hepatic form leads to recurrent metabolic decompensations often accompanied by elevated levels of liver transaminases (ELT) in blood, sometimes progressing to acute liver failure (ALF). Genetically, it is linked to the p.G229C variant in the *DLD* gene, which has been reported in the Ashkenazi Jewish and Arabic population. In this study, we analyzed phenotypic diversity, therapeutic management, and outcome in novel symptomatic individuals with hepatic DLDD identified by whole exome sequencing (*n* = 7) in Central Europe as well as in previously reported cases (*n* = 45). Fifty‐one of 52 DLDD patients carried the p.G229C variant (39 in a homozygous state). During decompensations, precipitated by febrile infectious disease or fasting, affected individuals presented with nausea, vomiting, abdominal pain, hepatomegaly, hypoglycemia, and lactic acidosis. In individuals homozygous for the p.G229C variant, neurologic manifestations were rare, whereas mild neurologic symptoms were found in individuals (*n* = 8) carrying a different *DLD* variant *in trans*. During decompensation, levels of specific plasma amino acids like citrulline or branched‐chain amino acids, and urinary organic acids, like 2‐oxoglutaric acid, were frequently elevated. However, known biomarkers—with the exception of lactate—were not consistently elevated during these episodes and typically normal in the interval, highlighting the usefulness of early genetic testing in all children with unexplained ELT or ALF to reduce the time to diagnosis. While there exists consensus for rescue therapy with intravenous glucose during decompensations and maintenance therapy with riboflavin, therapies with thiamine and antioxidants (e.g., N‐acetylcysteine) were reported to be useful in single individuals with recurrent decompensations.

## 1 Introduction

Dihydrolipoamide dehydrogenase (DLD) deficiency (DLDD; MIM 246900) is an autosomal recessive disease of mitochondrial metabolism which was first described in 1977. The index patient was a child who died at 7 months of age with progressive neurologic deterioration and lactic acidosis [1]. Thereafter, three distinct clinical phenotypes have been distinguished based on the predominant organ manifestation, that is, neurologic, myopathic, and hepatic disease variant [2]. The neurologic phenotype is characterized by developmental delay, muscular hypotonia, and infection‐triggered encephalopathy [2]. The myopathic phenotype leads to progressive muscular weakness, exertional fatigue, and rhabdomyolysis [3, 4]. The hepatic phenotype is marked by recurrence of elevated liver transaminase levels in blood (ELT), sometimes progressing to acute liver failure (ALF) with no or relatively mild neurologic symptoms [5]. The latter was first described in 1995 in a boy suffering from recurrent episodes of vomiting, lethargy, ALF, and hepatomegaly with lactic and ketoacidosis since birth, normal cognitive development, moderate motor impairment, and muscular hypotonia [6]. Later, the homozygous variant p.G229C in the *DLD* gene was identified to be causative for this hepatic phenotype of DLDD [5] which was shown to be a founder mutation among Ashkenazi Jews with a homozygosity rate of 1:35.000 newborns [5]. Subsequently, further individuals were reported also in the Arabic population [7]. Even though genotype–phenotype correlation in DLDD is not yet well understood, all reported individuals with the hepatic phenotype were homozygous or compound heterozygous for the pathogenic p.G229C variant [2, 8].

DLD is a core enzyme for mitochondrial energy and amino acid metabolism. It is the third catalytic subunit (E3) of three multienzyme complexes, namely the pyruvate dehydrogenase complex (PDC), the 2‐oxoglutarate‐dehydrogenase complex (KGDC) and the branched‐chain oxoacid‐dehydrogenase complex (BCKDC). Furthermore, DLD is part of the glycine cleavage system [2] and of the 2‐oxoadipate dehydrogenase complex, being essential for lysine metabolism [9]. The typical laboratory finding in all phenotypes is lactic acidosis, at least during decompensations. Further metabolic findings include hypoglycemia, elevated levels of plasma branched‐chain amino acids, allo‐isoleucine, citrulline, glutamine, and glutamate as well as elevated levels of 2‐oxoglutarate in urine [2]. However, these parameters are often normal in the interval and—with the exception of lactate—not always reliably altered during episodes of decompensation [9].

In this study, we systematically analyzed the clinical phenotype of subjects with hepatic DLDD, including 45 previously published cases and seven novel patients living in Central Europe. The aim of this study is to explore genotypes, the clinical course, and typical laboratory findings as well as therapeutic strategies.

## 2 Methods

### 2.1 Study Design, Identification of Affected Individuals and Clinical Characterization

Individuals with biallelic *DLD* variants were included from a whole exome sequencing (WES) study of patients with (recurrent) ALF of unknown etiology (for further details, see [10]) and individuals with recurrent ELT defined as at least three episodes with elevation of hepatic transaminases above the reference range (cohort 1). WES was performed as described before [10]: WES data was analyzed using an in‐house pipeline of the Technical University of Munich [11]. Genomic DNA was extracted from blood and WES performed [12]. Subsequently, sequencing reads were aligned to human genome‐build GRCh37/hg19 (UCSC Genome Browser) using the Burrows‐Wheeler Aligner (v.0.7.5a) [13]. Deletions, smart insertions, and single‐nucleotide variants (SNV) were detected with the Genome Analysis Toolkit [14] Copy number variants (CNV) were detected using ExomeDepth [15]. The medical history of all subjects was thoroughly reviewed. Data were collected in April 2024. The following terms of the Human Phenotype Ontology (HPO) were selected to describe the clinical phenotype: ELT (HP:0002910), ALF (HP:0006554), nausea and vomiting (HP:0002017), hepatomegaly (HP:0002240), hypoglycemia (HP:0001943), lactic acidosis (HP:0003128), triggered by febrile illness (HP:0025215), triggered by fasting (HP:0025212), cognitive impairment (HP:0100543), muscular hypotonia (HP:0001252), and post‐exertional symptom exacerbation (HP:0030973). Decompensation was defined by (a) clinical symptoms leading to medical consultation with (b) laboratory tests revealing at least one of the following: hypoglycemia, lactic acidosis, elevation of hepatic transaminases above the reference range.

All procedures followed were in accordance with the ethical standards of the responsible committee on human experimentation (institutional and national) and with the Helsinki Declaration of 1975, as revised in 2024. Informed consent to participate in the study was obtained from all individuals or, in the case of minors, from their parents. The study was approved by the ethical committee of the Technical University Munich and the ethical committee of the University Hospital Heidelberg. To search for published cases (cohort 2), PubMed was screened using the mesh term “Dihydrolipoamide Dehydrogenase Deficiency AND liver” and “Dihydrolipoamide Dehydrogenase Deficiency AND hepatic,” furthermore the references of all publications were screened for further relevant publications in August 2024. Inclusion criteria were genetically confirmed DLDD with a clinically apparent primarily hepatic manifestation.

All tables and figures were designed using R, Microsoft Excel, and Microsoft Power Point. The Wilcoxon test was used for comparing the laboratory values during crises and in the interval. For group differences between individuals with a homozygous p.G229C variant and a compound heterozygous p.G229C variant with different variants in *trans*, the two‐sample *t*‐test was used for mean age and the Chi squared test for occurrence of clinical symptoms.

## 3 Results

### 3.1 Description of Individuals With the Hepatic Variant of DLDD Identified by WES (Cohort 1)

Seven individuals from six families with pathogenic variants in *DLD* and a hepatic phenotype were identified, of whom six individuals were identified via WES. Five individuals carried the homozygous p.G229C variant, and one of them (DLD‐4) was compound heterozygous for the protein variant p.G229C and the variant c.1046+5G>T; p.I293_N349del. The seventh individual (DLD‐7) had a WES with copy number variation (CNV) analysis done and was heterozygous for the p.G229C variant, with no second pathogenic variants in *trans* being identified. Other causes for liver disease were not identified. Functional analyses, including RNA sequencing, immunofluorescence microscopy, and PDC activity, were hence performed due to high clinical suspicion for DLDD, revealing a monoallelic transcription of the *DLD* gene, reduced protein expression of DLD, and reduced PDC activity in the patient's fibroblasts, thus confirming the diagnosis of DLDD (see Figure S1). Six individuals were female. Age at last report ranged between 4.5 and 24 years (median 7.9 years); all individuals were alive in April 2024. They lived in Central Europe (3/7 Germany, 1/7 Austria, 2/7 Switzerland, 1/7 Hungary), and most families were of (Ashkenazi) Jewish or Arabic origin (5/7), while one was of German and one of Romani origin. All individuals had recurrent decompensations manifesting with nausea and vomiting and ELT; 5/7 presented with ALF (in 4/5 recurrent). Hepatomegaly during decompensations was present in 5/7 individuals, hypoglycemia in 5/7 individuals, and lactic acidosis in 6/7 individuals. First decompensations occurred between day 2 and 3.4 years and were precipitated by, if known, febrile illness (7/7), fasting (3/5), and less frequently by vaccination (DLD‐4, DLD‐7), psychosocial stress (DLD‐1) and non‐adherence to therapy (DLD‐1). A single individual (DLD‐7) had mild delay of speech and post‐exertional fatigue, while the remaining six individuals did not develop neurologic or myopathic signs or symptoms until the last visit. Therapeutic management varied, ranging from no permanent treatment (*n* = 1), only riboflavin supplementation (*n* = 2) to treatment with a combination including riboflavin (*n* = 4), thiamine (*n* = 4), N‐acetylcysteine (NAC) (*n* = 3), lipoic acid (*n* = 1), vitamin E (*n* = 1), coenzyme Q10 (*n* = 1), carnitine (*n* = 2), MCT (*n* = 1), corn starch (*n* = 1), late meals with complex carbohydrate meals (*n* = 2), sodium bicarbonate (*n* = 1), vitamin K (*n* = 1), and intravenous immunoglobulins (*n* = 1) (Table 1, Supporting Information, Figure S2).

**TABLE 1:** Genetic and phenotypic characterization and therapeutic approach of cohort 1 of individuals with hepatic DLDD.

ID	DLD‐1	DLD‐2	DLD‐3	DLD‐4	DLD‐5	DLD‐6	DLD‐7
Sex	M	F	F	F	F	F	F
Nationality	Germany	Germany	Hungary	Austria	Switzerland	Switzerland	Germany
Descent	Lebanese	Turkish	Romani	Ashkenazi‐Jewish	Jewish	Jewish	German
Age at last visit/report	13.4 ys	24 ys	12 ys	4.5 ys	7.9 ys	4.8 ys	7.3 ys
Alive at time of database closure?	Y	Y	Y	Y	Y	Y	Y
DLD variant, allele 1	c.685G>T; p.G229C	c.685G>T; p.G229C	c.685G>T; p.G229C	c.685G>T; p.G229C	c.685G>T; p.G229C	c.685G>T; p.G229C	c.685G>T; p.G229C
DLD variant, allele 2	c.685G>T; p.G229C	c.685G>T; p.G229C	c.685G>T; p.G229C	c.1046+5G>T; p.I293_N349del	c.685G>T; p.G229C	c.685G>T; p.G229C	monoallelic expression
Characterization of decompensations							
Number of decompensations	30	9	4	6	6	3	29
Age at first decompensation	3.4 ys	3 ys	1.5 ys	2 days	3 days	3.3 ys	7 days
Age at most severe decompensation	11.1 ys	16 ys	3 and 7 ys	2 days	3 days	3.3 ys	5.9 ys
Age at last decompensation	13.4 ys	21 ys	9 ys	2.9 ys	6.7 ys	4.8 ys	7 ys
Elevated hepatic transaminases	Y	Y	Y	Y	Y	Y	Y
Acute liver failure	N	Y	N	Y	Y	Y	Y
Nausea and vomiting	Y	Y	Y	Y	Y	Y	Y
Hepatomegaly	Y	Y	Y	Y	N	N	Y
Hypoglycemia	Y	N	Y	Y	Y	N	Y
Lactic acidosis	Y	Y	N	Y	Y	Y	Y
Decompensations triggered by febrile illness	Y	Y	Y	Y	Y	Y	Y
Decompensations triggered by fasting	N	n.a.	N	Y	Y	n.a.	Y
Others symptoms during decompensation	Abdominal pain	Hepatic encephalopathy	Acute pancreatitis	Muscular hypotonia	None	None	Apathy, abdominal pain, seizures
Other triggering factors	Stress, incompliance to medication			Vaccination			Meningococcus B vaccination
Extrahepatic symptoms							
Cognitive impairment reported*	N	N	N	N	N	N	N
Muscular hypotonia	N	N	N	During decompensations	N	N	N
Post‐exertional symptom exacerbation	N	N	N	N	N	N	Y
Others	Subcutaneous hemangioma of the right forehead, hyperopia						Development delay of speech and language, frequent infections
Therapy							
Maintenance therapy, daily dosages							
B_1_ (thiamine)	Y (3 × 100 mg)	N	N	Y (3 × 100 mg)	N	N	Y (3 × 100 mg)
B_2_ (riboflavin)	Y (3 × 50 mg)	Y (2 × 20 mg)	N	Y (2 × 50 mg)	Y (2 × 100 mg)	Y (2 × 100 mg)	Y (3 × 100 mg)
NAC	N	N	N	Y (150 mg/kg/d)	N	N	Y (180 mg/kg/d)
Ketogenic diet	N	N	N	N	N	N	Y (initiation planned for 2025)
Carnitine	N	Y (3 × 1 g)	N	Y (3 × 330 mg)	N	N	N
Lipoic acid	N	N	N	Y (2 × 100 mg)	N	N	N
MCT	N	N	N	N	N	N	Y (4 × 6 g)
Others		Vitamin E (2 × 400 mg), coenzyme Q_10_ (60 mg)		High carbohydrate diet, sodium bicarbonate, IVIG monthly until the age of 3.3 years			Starch (2 × 20 g) (7 months—4.7 years) and late meal (from 4.7 years on); 4–17 months vitamin K peroral
Rescue therapy							
Intravenous glucose	Y	Y	Y	Y	Y	Y	Y
NAC (intravenous)	Y (300 mg/kg)	N	N	N	N	N	Y (300 mg/kg)

*Note:* Cognitive impairment reported*: No standardized neuropsychological tests performed; assumption based on the clinical evaluation of the caring physician and the reported attendance of a regular school/kindergarten.

Abbreviations: DLD(D), dihydrolipoamide dehydrogenase (deficiency); F, female; ID, identification number; IVIG, intravenous immunoglobulins; M, male; MCT, medium chain triglycerides; N, no; n.a., not available; NAC, N‐acetylcysteine; Y, yes; ys, years.

### 3.2 Review of All Individuals With the Hepatic Variant of DLDD Including WES Cohort (Cohort 1) and all Previously Published Individuals (Cohort 2)

So far, 45 individuals with biallelic pathogenic variants in *DLD* and a predominantly hepatic phenotype have been reported (cohort 2) [5, 6, 7, 8, 9, 16, 17, 18, 19, 20, 21, 22, 23, 24, 25, 26, 27, 28, 29] (for details, see Table S1). Together with our seven novel patients, 52 individuals (26 female, 26 male) were hence included in this study.

All individuals but one carried at least one p.G229C variant, 39 of whom were in homozygous state. Of the 13 remaining individuals, six carried the variant p.Y35X in *trans*, six individuals had a second compound heterozygous variant only reported in one individual each, and one had a monoallelic expression of the allele giving rise to the p.G229C variant (see above, Figure S1). The only individual without the p.G229C variant had a homozygous p.D479V variant [8], common in the Bedouin population and typically associated with a severe neurologic phenotype.

Age at last clinical assessment ranged between 3 months and 47.7 years (median 15.5 years), and age at onset between first day of life and 34 years (median 3 days). Seven individuals died between the ages of 0.3 years and 47.7 years (median 6.5 years). Presumed cause of death was severe metabolic decompensations with or without ALF in three, and sepsis in one. One further individual had severe ALF with multiorgan failure requiring high urgency liver transplantation. Recovery after transplantation was poor, and the individual died from sepsis shortly after [28]. Individuals with a compound heterozygous variant p.G229C and a different variant in *trans* were significantly younger at onset than individuals with a homozygous p.G229c variant; there was no difference regarding survival (see Table 2).

**TABLE 2:** Phenotypic differences between individuals with a *DLD* homozygous p.G2229C variant and a compound heterozygous p.G229C variant with different variants *in trans*. *p*‐values were calculated using a two‐sample *t*‐test assuming equal variances for mean age and Chi squared test for the others. Absolute numbers are given in brackets; *p*‐values showing significant differences are marked with a star.

	Individuals with a homozygous p.G229C variant	Individuals with a compound heterozygous p.G229C variant and a different variant *in trans*	*p*
Number of individuals	39	12	
Mean age at manifestation (years)	4.60 (*n* = 33)	0.007 (*n* = 9)	0.0251*
Alive at time of report (*n*)	84.62% (33/39)	100% (12/12)	0.2478
Elevated hepatic transaminases	100% (37/37)	100% (12/12)	—
Acute liver failure	84.21% (16/19)	100% (5/5)	0.4487
Nausea and vomiting	100% (28/28)	100% (4/4)	—
Hepatomegaly	85.71% (18/21)	100% (4/4)	0.4203
Hypoglycemia	70.81% (17/24)	90% (9/10)	0.2299
Lactic acidosis	93.33% (28/30)	100% (10/10)	0.5587
Cognitive impairment	3.03% (1/33)	16.67% (2/12)	0.1049
Muscular hypotonia	6.90% (2/29)	71.43% (5/7)	0.0001*
Post‐exertional symptom exacerbation	55.56% (10/18)	50% (1/2)	0.8809
Exclusive hepatic phenotype	64.10%	33.33%	0.0520
Neurologic symptoms	12.82%	66.67%	0.0002*
Hepatic encephalopathy	48.72%	41.67%	0.6686

**FIGURE 1:**
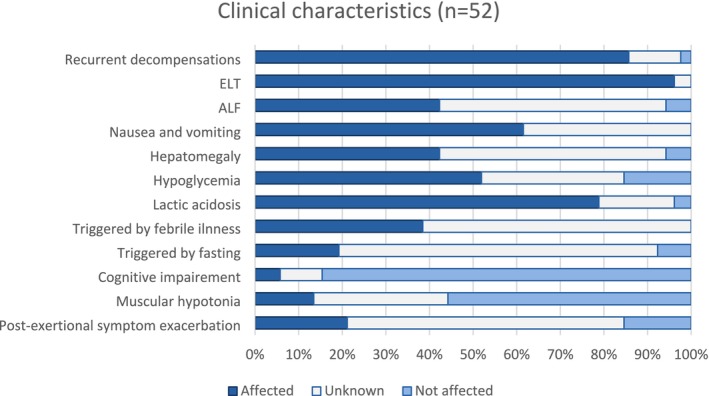
Clinical characteristics of currently known individuals with a primarily hepatic phenotype of DLDD. ALF, acute liver failure; DLDD, Dihydrolipoamide dehydrogenase deficiency; ELT, elevated liver transaminases.

All individuals had hepatic involvement with either ELT or ALF. Nausea, vomiting, and hepatomegaly were characteristic clinical findings, and hypoglycemia and lactic acidosis were characteristic biochemical findings (for details see Figure 1, Table 1 and Table S1). There were no significant differences in those clinical findings between individuals with a homozygous p.G229C variant and a compound heterozygous p.G229C variant with a different variant in *trans* (see Table 2). Other frequently reported symptoms during decompensation included abdominal pain (16 individuals), hepatic encephalopathy or somnolence/apathy/lethargy (23 individuals), seizures (two individuals), rhabdomyolysis (two individuals), muscle weakness/hypotonia (six individuals), sometimes with loss of deep tendon reflexes (three individuals). Apart from the typical triggering factors, febrile illness and fasting, two individuals each also had decompensations triggered by excessive meals [21], tiredness [21] and vaccination (DLD‐4 and DLD‐7, both cohort 1). With regards to the neurologic and myopathic phenotype outside the decompensations, cognitive impairment was rare (3/47 individuals), 7/36 individuals had muscular hypotonia, and 11/19 had post‐exertional symptom exacerbation (see Figure 1). Other reported symptoms were behavioral problems (five individuals) and developmental disorders (six individuals). With regards to the different genotypes (homozygous p.G229C and compound heterozygous p.G229C with different variants in *trans*), neurologic symptoms and muscular hypotonia were significantly more often reported in the compound heterozygous group (see Table 2).

Liver biopsy was performed five times in three individuals in cohort 1 (DLD‐2, DLD‐3 and DLD‐7) between the ages of 7 months and 21 years (mean 4 years). Liver biopsies were mostly done during diagnostic work‐up before the molecular diagnosis was established or for follow‐up of liver remodeling. In addition, seven biopsies in five individuals were reported in cohort 2 between the ages of 3.5 and 16 years (mean 8.5 years) [21, 22, 27, 28]. Results ranged from normal light microscopy (five biopsies) with two showing accumulation of lipid droplets in electron microscopy; microvesicular steatosis (two biopsies) with portal inflammation in one; fatty, acute necrosis and Reye‐like mitochondria in electron microscopy, and cholestasis as well as fibrosis reported in a follow‐up biopsy in one individual; mild fibrosis and steatosis in two biopsies from the same individual; and extensive predominantly periportal necrosis and canalicular cholestasis in another affected patient.

Different treatment schemes included thiamine (15 individuals), riboflavin (26 individuals), carnitine (13 individuals), lipoic acid (3 individuals), dichloroacetate (DCA) (5 individuals), coenzyme Q_10_ (4 individuals), biotin (5 individuals), sodium‐succinate (1 individual), and restriction of branched‐chain amino acids (1 individual) with no clear conclusion concerning improvement. Single case reports or case series describe clinical or laboratory improvement with DCA, thiamine, and carnitine [5, 18], and others with coenzyme Q and biotin [7]. Five individuals did not receive specific treatment between crises [8, 25], and three did not respond to therapy [21]. One individual was described as having thiamine deficiency upon administration of dichloroacetate [5]. In recent publications, the mostly used treatments were thiamine, riboflavin, and carnitine [8, 25]. One individual [26] underwent liver transplantation, though with a fatal outcome. Emergency management of acute metabolic and hepatic crises was not reported in detail in the literature.

### 3.3 Biochemical Biomarkers During Crises and in the Interval

**FIGURE 2:**
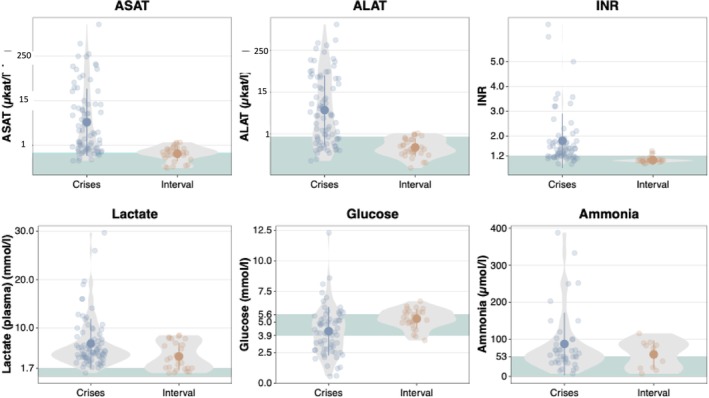
Distribution of maximum levels of AST and ALT (both with logarithmic scale), INR, lactate, ammonia, and serum minimal values of glucose during crises and in the interval. Approximate reference values are given in green for comparison, though those are not age‐, gender‐, and laboratory‐adjusted. **p ≤ 0.01; ****p ≤ 0.0001 by Wilcoxon test. ALT, alanine transferase; AST, aspartate transferase; INR, international normalized ratio.

Taken together from all individuals with DLDD (cohort 1 and cohort 2), AST, ALT, INR, and lactate were significantly elevated during crises in comparison to the interval, and glucose was significantly decreased (Figure 2). The median values during crises were AST 1.72 μkat/l (range 0.33–872.83 μkat/l; ref. < 0.65 μkat/l, i.e., 39 U/L), ALT 2.79 μkat/l (range 0.18–694.67 μkat/l; ref. < 0.83 μkat/l, i.e., 50 U/L), INR 1.4 (range 0.86–3.7; ref. < 1.2), lactate 4.79 mmol/L (range 0.7–29.7 mmol/L; ref. < 1.7 mmol/L), ammonia 93 μmol/L (range 17–387.7 μmol/L; ref. < 53 μmol/L), and glucose 4.16 mmol/L (range 0.6–12.3 mmol/L; ref. 3.9–5.6). In the interval, median values were AST 0.68 μkat/l (ref. < 0.65 μkat/l, i.e., 39 U/L), ALT 0.44 μkat/l (ref. < 0.83 μkat/l, i.e., 50 U/L), INR 1.01, lactate 3.5 mmol/L, ammonia 77 μmol/L, and glucose 5.33 mmol/L. For exact values, see Tables S2 (WES cohort) and S3 (previously published individuals).

### 3.4 Amino Acid Concentrations in Plasma

In 28/45 individuals of cohort 2, for whom test results of plasma amino acids were reported, increased concentrations for leucine, isoleucine, valine, alanine, glutamine, and citrulline were frequently reported (see Table 2 and Tables S4 and S5). In cohort 1 (Table 2, Table S4), citrulline was the most frequently elevated plasma amino acid during crises, followed by leucine, glutamine, glutamate, and valine. However, in 4/30 crises, plasma amino acid concentrations remained in the reference range, while the concentrations of single amino acids were elevated in 8/30 crises. In the interval (21 analyses in four individuals), amino acid profiling was unremarkable in 6/21 analyses, while in 15/21 analyses concentrations of single amino acids, mostly glycine and glutamine, were abnormal.

**FIGURE 3:**
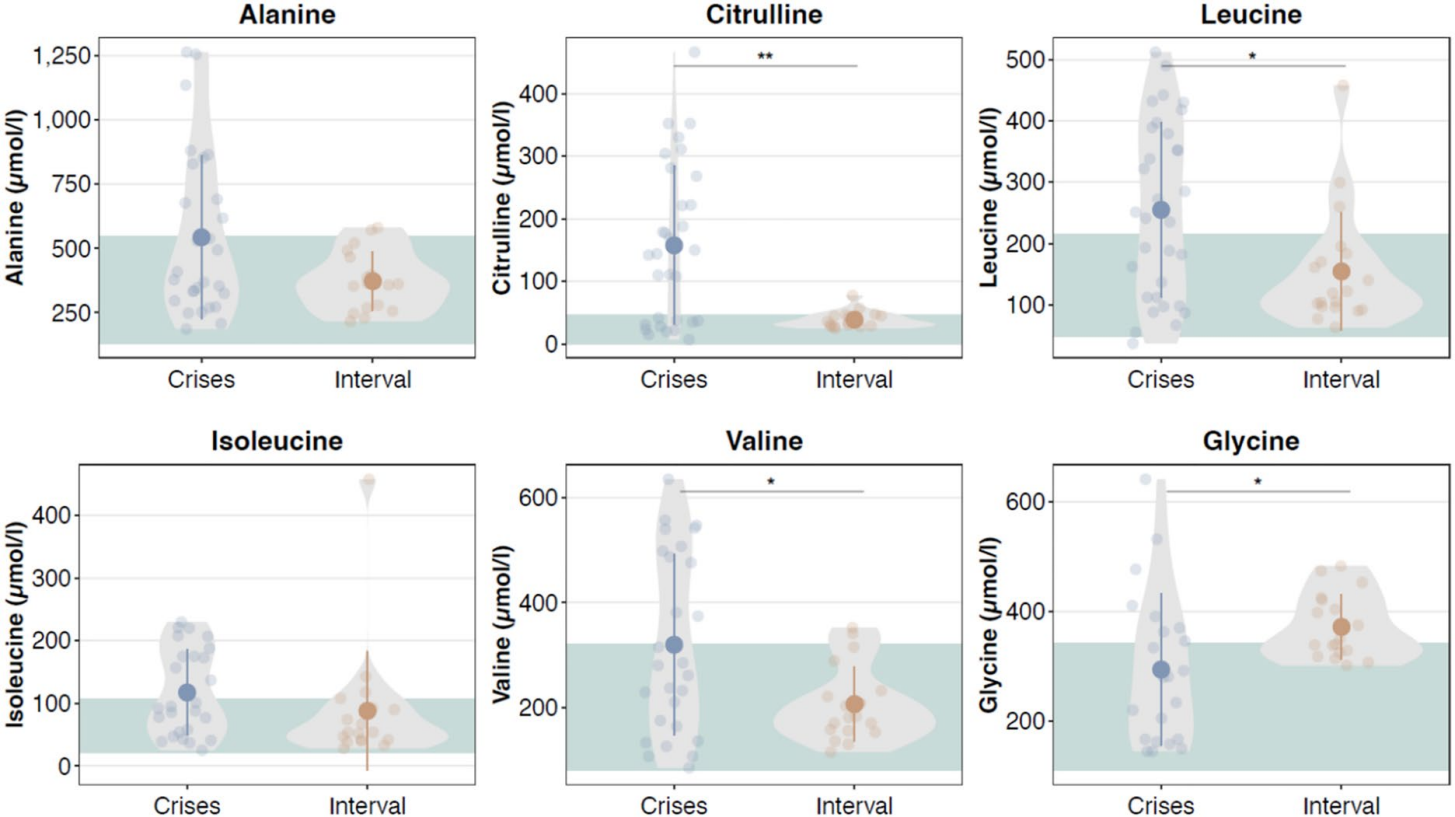
Comparison of plasma concentrations of alanine, citrulline, leucine, isoleucine, valine, and glycine during crises and in the interval. Approximate reference ranges are given in green. *p ≤ 0.05; **p ≤ 0.01 by Wilcoxon test.

When comparing plasma amino acid levels of all individuals with DLDD in the interval and during crises, citrulline, leucine, and valine are elevated during crises; whereas glycine is higher in the interval (see Figure 3).

### 3.5 Organic Acid Concentrations in Urine

In the WES cohort of DLDD (cohort 1), urinary concentrations of organic acids were analyzed in six individuals during 21 crises and 15 times in five individuals in the interval. Test results were compared to the results of 23 organic acid analyses reported for the literature cohort (cohort 2). While the 2‐oxoglutaric acid level was most frequently increased in the literature cohort during decompensations, increased urinary concentrations for 2‐hydroxyglutaric acid, glutaric acid, and 2‐hydroxyadipic acid were found to be most common in the WES cohort (see Table 3 and Tables S4 and S5 for exact values). In the WES cohort, concentrations of urinary organic acids were found to be in the reference range for six of 21 analyses during crises and 14 of 15 analyses in the interval.

**TABLE 3:** Elevation of plasma amino acid levels and urinary organic acid levels in our cohort and the published literature during crises and in the interval.

	DLD‐1		DLD‐2		DLD‐3		DLD‐4		DLD‐5	DLD‐6	DLD‐7			All together	
	C	I	C	I	C	I	C	I	C	C	C	I	Cohort 2	C	I
Amino acid															
N*	10	6	2	0	1	1	2	4	6	2	7	10	28	58	21
Alanine	3↑						1↑	2↑		1↑			8↑	13↑	2↑
Glutamate	7↑						1↑	3↑		2↑			3↑	13↑	3↑
Glutamine	9↑	1↑					1↑	2↑	1↑			3↑	8↑	19↑	6↑
Citrulline	9↑	2↑						2↑	6↑	2↑			8↑	25↑	4↑
Leucine	7↑						1↑	2↑	5↑	2↑	1↓	1↑	9↑+	24↑+, 1↓	3↑
Isoleucine	6↑							2↑		1↑		2↑	9↑+	16↑+	4↑
Valine	7↑		1↑		1↓			2↑		1↑		1↑	8↑+	17↑+, 1↓	3↑
Lysine	1↓						1↑			1↓	1↓		1↑1↓	2↑, 4↓	
Proline	3↑						1↑	1↑					1↑	5↑	1↑
Glycine	3↑	4↑					1↑	1↑			2↑	4↑	1↑	7↑	8↑
Alloisoleucine	3↑									2↑		1↑	4↑	9↑	1↑
Organic acid															
N*	9	6	3	2	1	1	4	1	1	3	0	5	23	44	15
Ketone bodies	1↑												4↑	5↑	
Fumaric acid													3↑	3↑	
Succinic acid							1↑						2↑	3↑	
Glutaric acid	3↑						1↑	1↑	1↑	3↑			4↑	12↑	1↑
2‐Hydroxyglutaric acid	3↑						2↑		1↑	3↑			3↑	12↑	
2‐Oxoglutaric acid							1↑	1↑	1↑				9↑+	11↑+	1↑
5‐Oxoproline acid	1↑													1↑	
2‐Hydroxy‐3‐methylvaleric acid													1↑	1↑	
3‐Methylglutaconic acid													1↑	1↑	
3‐Methylglutaric acid															
2‐Oxoisocaproic acid	2↑													2↑	
2‐Hydroxyisovaleric acid	3↑									3↑			2↑	8↑	
2‐Oxo‐adipic acid	1↑		1↑							1↑			5↑	8↑	
2‐Hydroxy‐adipic acid	4↑		2↑						1↑				6↑	13↑	
Adipic acid	3↑								1↑	2↑			2↑	8↑	
3‐Hydroxy‐sebacic acid	2↑								1↑					3↑	

Note: N* Number of crises with measured amino acids/organic acids and number of measurements in the interval. + plus an unknown number of individuals within a cohort of 8 additional individuals with an infrequent elevation of each amino acid level and an infrequent elevation of each organic acid level in a cohort of 11 additional individuals.

Abbreviations: C, crisis; I, interval; ↑ increased; ↓ decreased.

## 4 Discussion

In this observational, multicenter, and literature study, we studied the clinical and biochemical manifestation, therapy, and outcome in a cohort of 52 individuals with the hepatic form of DLDD, seven of whom have not been published before.

### 4.1 Clinical Manifestation

#### 4.1.1 Genotype/Phenotype Correlation and Possible Mechanisms

There is a homogenous clinical presentation of hepatic DLDD with recurrent crises characterized by lactic acidosis, vomiting, abdominal pain, hypoglycemia, and ELT or ALF triggered by infections or fasting; however, crises can be heterogeneous even within the same individual. Disease onset is heterogeneous throughout life, but most frequently found within the first 3 years. All but one of the 52 individuals evaluated in this study carried the p.G229C variant, 39/51 of them in a homozygous state. However, individuals with homozygosity for the p.G229C variant can remain asymptomatic [8], or have metabolic decompensations with lactic acidosis and hypoglycemia without any sign of liver involvement [7, 8, 28]. Thus, there is a great heterogeneity among individuals with the same homozygous variant. Environmental factors, further genetic modifiers, and potentially specific pathogens triggering decompensations might contribute to this heterogeneity.

While 29/52 (56%) had an exclusively hepatic manifestation without neurologic or myopathic symptoms in the interval, mild neurologic symptoms were frequently reported in individuals with one p.G229C variant together with another variant in *trans*. While a differentiation between primary neurological involvement and secondary involvement due to hepatic encephalopathy remains impossible, a mild neurologic manifestation in hepatic DLDD, especially in individuals with only one p.G229C variant, is likely. Mild neurologic and myopathic symptoms in those individuals are expressions of an overlap between the three DLDD phenotypes: hepatic, neurologic, and myopathic.

Via dysfunction of multiple enzyme complexes in DLDD, energy metabolism is profoundly disturbed regarding glucose, protein, and—via dysfunction of the Krebs cycle—also fatty acid metabolism. A reduced activity of DLD, PDC, and KGDC in fibroblasts and muscle tissue in individuals with the p.G229C variant was described multiple times [5, 6, 7, 18]. However, the DLD/PDC activity in fibroblasts seems to be less decreased than in individuals with more severe neurologic manifestations of DLDD [30, 31]. Similarly, ATP synthesis is reduced in DLDD [31] with higher ATP production rates in p.G229C mutants compared to other genotypes that give rise to more severe neurologic manifestations. This is in line with our findings that a heterozygous p.G229C variant is associated with mild neurologic manifestation, and potentially the presence of one p.G229C variant may prevent the development of a severe neurologic manifestation.

#### 4.1.2 Hepatic Manifestation

Individuals with DLDD typically present with ELT or ALF. In contrast to other mitochondrial disorders linked to ALF, such as TRMU or DGUOK deficiency, there is no cholestatic pattern [32, 33]. On the other hand, mean hepatic transaminase activity levels as a marker for hepatocytolysis were rather low compared to a cohort of individuals with ALF of unknown origin: AST 32.32 μkat/l (1.72 μkat/l in DLDD; ref. < 0.65 μkat/l 39 U/L) and ALT 15.12 μkat/l (2.78 μkat/l in DLDD; ref. < 0.83 μkat/l 50 U/L) [10]; the same holds true for INR: median INR 3 (1.4 in DLDD) [10]. Hence, DLDD leads to a less severe hepatocytolysis and liver function impairment. Whether the frequency of crises decreases with age cannot be determined due to the small sample size and heterogeneous group; however, some individuals only develop ELT/ALF in adulthood [5]. Liver enlargement was evident in most individuals with DLDD either by sonogram or physical examination. Liver biopsies were rarely performed and did not reveal a typical pattern: only 2/8 individuals with available liver biopsy had signs of progressive liver disease in the form of mild fibrosis, while in none of the biopsies were signs of liver cirrhosis observed.

ELT or ALF in DLDD emerge with acute metabolic crises; however, not every metabolic decompensation is associated with a hepatic crisis. As crises are typically triggered by (viral) infections, the impact of those infections on the occurrence of ELT is possible. Furthermore, myopathic involvement could contribute in part to AST/ALT elevation. In the p.G229C variant, an increased reactive oxygen species (ROS)‐generation activity via superoxide was shown, which was also more sensitive to an acidic pH [30]. This could contribute to liver dysfunction in hepatic DLDD. Higher ROS activities are linked to hepatic dysfunction, for example, in acetaminophen intoxication [34]. We hypothesize that a certain threshold of metabolic decompensation is necessary for hepatic function disturbance, potentially via lactic acidosis and subsequent increased ROS generation. Early therapy with glucose infusion (or spontaneous increased oral glucose intake) and antioxidant therapy (e.g., N‐acetylcysteine) might protect from hepatic decompensations.

### 4.2 Laboratory Findings

#### 4.2.1 Lactic Acidosis and Hypoglycemia

Because of the deficient PDC, elevation of lactate levels was expected during metabolic decompensation. However, in our cohort, one individual never had increased levels of lactate in line with previously reported cases [21, 27]. This might be due to rapid normalization of lactate levels upon glucose infusion or delayed measurement during an acute episode in the individuals, as metabolic decompensation without any lactate level elevation seems unlikely. Hypoglycemia, though often seen during decompensations, is not always present. Hyperglycemia is less commonly found and may reflect high glucose intake, early postprandial state, or the administration of IV glucose.

#### 4.2.2 Metabolic Markers

During metabolic crises, elevations of the plasma amino acid levels citrulline, leucine, isoleucine, valine, and alanine as well as glutamine and glutamate were seen regularly. In urine, 2‐oxoglutarate concentrations were described to be elevated during metabolic crises, whereas in cohort 1 this was not common and, in the interval, urinary organic acid levels were often normal. Elevations of branched‐chain amino acid levels in plasma and 2‐hydroxy‐isovaleric acid as well as 2‐oxoisocaproic acid levels in urine can be well explained by the deficiency of the BCKDC, as are elevations of urinary 2‐oxoglutarate levels by the deficient KGDC. While elevated glutamate and glutamine levels are likely secondary to the increase of 2‐oxoglutarate, increased alanine levels are due to the dysfunction of the PDC. Data on the pyruvate‐to‐lactate ratio, which might give further insight into PDC involvement, were non‐conclusive in the literature and not available in cohort 1. Increased glycine levels are explained by the deficiency of the glycine cleavage system; further studies are needed to understand why in cohort 1 plasma glycine levels were rather frequently elevated in the interval but only rarely during crises.

According to Wongkittichotte et al. [9] also lysine metabolism is also affected due to a deficient 2‐oxoadipate dehydrogenase complex. In their study of seven individuals with DLDD (three with the hepatic form), reduced lysine levels were reported to be the most sensitive amino acid marker. However, among the three individuals with the hepatic form, lysine levels were only reduced in one. In cohort 1, lysine levels in plasma were reduced in 3/7 individuals during metabolic crises. Furthermore, we found elevations of 2‐hydroxyadipic acid and 2‐oxo‐adipic acid levels in urine, pointing to a deficient 2‐oxoacid dehydrogenase complex (OADC), overall supporting the hypothesis of a potentially impaired 2‐oxoadipate dehydrogenase complex in DLDD.

Other plasma amino acid and urinary organic acid level elevations are likely secondary to deficiency of the intermediate metabolism with a deficient PDC. Elevation of citrulline levels can be explained by deficient PDC resulting in reduced production of oxaloacetate as a result of insufficient tricarboxylic acid cycle and/or insufficient gluconeogenesis and thus aspartate depletion with interference of argininosuccinate formation as suggested by Haviv et al. [35]. In two studies (Haviv et al. [35] and Siri et al. [29]), plasma citrulline level was suggested as a good marker for detecting DLDD, even during the newborn screening. However, citrulline is not commonly elevated in PDC deficiency alone and within all crises documented in cohort 1, citrulline levels were elevated in less than half the crises and never elevated in 3/7 individuals. Values from the newborn screening are known for 3/7 individuals; the citrulline level was elevated in one and normal in two. Thus, citrulline elevation is not a reliable biomarker for detection of DLDD.

Conclusively, plasma amino acid and urinary organic acid level elevations in hepatic DLDD are heterogeneous due to the many different pathways affected. As neither a single amino acid level nor a single organic acid level is always pathological during crises, there is no single powerful diagnostic biomarker, but a biochemical pattern as described above. The resulting mitochondrial energy deficit with lactic acidosis might be the common effector pathway leading to elevated ROS generation [30] and hence to liver cell damage. Additional study are needed to shed further light on the pathomechanism.

#### 4.2.3 Diagnostic Workup

As laboratory parameters are usually normal in the interval, we suggest that in all individuals with unclear ELT (or ALF), glucose and lactate levels should be measured early during decompensation. In children with repeated decompensations triggered by febrile illness or by fasting, metabolic workup should include amino acid levels in plasma and organic acid levels in urine. Physicians should be aware that values can normalize quickly during glucose infusions, and sampling should be performed before treatment initiation. As the metabolic workup can remain unspecific and DLDD represents an important differential diagnosis in individuals with (recurrent) ELT or ALF, especially when lactic acidosis and hypoglycemia are present, genetic diagnostic (e.g., whole exome or genome sequencing) should be considered early [10].

### 4.3 Therapy and Prevention

We recommend early antipyretic treatment in case of febrile infections although there is no evidence of temperature‐induced decompensations. Especially with impending acute liver failure, non‐steroidal anti‐inflammatory drugs should be avoided due to the inhibition of platelet aggregation. Therefore, metamizole or a combination of acetaminophen together with NAC might be the most suitable options. Two individuals had decompensations following a vaccination, most likely due to mild temperature elevation and reduced appetite with consecutive catabolism in the post‐vaccination course. Nevertheless, we recommend routine vaccinations in all affected children, as febrile infections frequently lead to decompensations although. Fasting and catabolism should be avoided, especially during intercurrent illness.

Emergency management with high dose intravenous glucose administration is recommended in individuals with DLDD and metabolic crisis, as it reverses the catabolic state leading to normalization of lactic acidemia. Blood lactate should be carefully monitored as it may rise due to the deficient PDC. Interestingly, this was not seen regularly upon glucose infusion in our cohort.

#### 4.3.1 Maintenance Therapy With Cofactors (Thiamine and Riboflavin)

Maintenance therapy in DLDD is heterogeneous: most individuals receive riboflavin as part of the maintenance therapy. The active derivate of riboflavin (Flavin adenine dinucleotide (FAD)) is a cofactor of DLD [36] and supports electron transfer to the respiratory chain. The second most frequently used drug was thiamine, which together with DLD/E3 is a cofactor of the PDC, the BCKDHC, and the KGDC. While in one study there was no improvement of the energetic state in p.G229C homozygous cells with riboflavin and thiamine [31], in a *Caenorhabditis elegans* and a zebrafish model, maintenance therapy with thiamine was beneficial [37, 38]. In the myopathic DLDD, riboflavin was shown to have positive effects on energy production, cellular ATP content, and lowered oxidative damage, potentially due to direct redox effects or due to its chaperon‐like ability to correct protein folding [4]. Those functions might contribute to positive effects in hepatic DLDD. In cohort 1, beneficial effects of riboflavin with a decreased frequency and/or severity of metabolic crises in single individuals were reported; the same holds true for thiamine. In support of this, crises were triggered by noncompliance with medication in one individual. On the other hand, some individuals have no further crises without maintenance therapy. Further studies are needed to accurately assess the benefit of single cofactors.

#### 4.3.2 Antioxidant Therapy

An increased ROS‐generating activity sensitive to a decreased pH in the p.G229C variant [30] points to potential benefits from antioxidant therapy. Interestingly, acetaminophen intoxication leads to ALF via mitochondrial dysfunction and ROS generation, and the antioxidant N‐acetylcysteine (NAC) is an approved therapy [39]. It was used in three affected individuals, either as maintenance therapy or only during metabolic decompensation for prevention of ALF. Therapy was reported to be beneficial in those individuals; however, data is sparse and there is a need for further studies before drawing conclusions. Of note, NAC is not generally beneficial in children with non‐acetaminophen induced ALF. In a large cohort, NAC did not improve survival and even reduced liver transplantation‐free 1‐year survival [40]. We hypothesize that a subgroup of ALF patients presenting with mitochondrial dysfunction may benefit from such an antioxidant therapy.

Apart from NAC, other antioxidant therapies were used in single individuals, namely lipoic acid, coenzyme Q_10_, and vitamin E. Only for lipoic acid, there is a single case report with positive effects [6, 20], but with mixed results in vitro [31, 41].

#### 4.3.3 Liver Transplantation

Liver transplantation was performed in one single case with hepatic DLDD during a multiorgan failure episode with a fatal outcome shortly after the procedure [26]. However, as some individuals had fatal metabolic crises with ALF, liver transplantation might be considered in severely affected individuals; especially as neurologic outcome is generally promising.

## 5 Conclusion

The hepatic form of DLDD is caused by a homozygous or compound heterozygous *DLD*‐p.G229C variant. It typically leads to recurrent metabolic crises triggered by infection or fasting, with vomiting, abdominal pain, ELT/ALF, lactic acidosis, and hypoglycemia. Whereas individuals with a homozygous p.G229C variant typically do not present neurologic symptoms, mild neurologic involvement is typical in individuals with a compound heterozygous p.G229C variant. While hepatic transaminase levels and INR are usually not drastically elevated, decompensation can lead to life‐threatening, severe acute liver failure. As metabolic biomarkers are not consistently present, genetic diagnostics should be initiated early in the diagnostic work‐up. For treatment, there is consensus for a maintenance therapy with riboflavin, and positive effects of additional therapy in single individuals with thiamine and antioxidants (e.g., NAC) were observed; further studies are needed to draw conclusions. Crises should be managed strictly with glucose infusion during acute decompensations under lactate monitoring to ensure an anabolic state.

## Author Contributions

Dominic Lenz, Christian Staufner, Georg F. Hoffmann, Stefan Kölker, and Nicole Hammann were responsible for the conception and the design of the study. Data acquisition was performed by Nicole Hammann, Dominic Lenz, Christian Staufner, Antal Dezsőfi‐Gottl, Johannes Häberle, Valérie McLin, Norman Junge, Vassiliki Konstantopoulou, Ekkehard Sturm, Daisy Rymen, and Christoph Slavetinsky. Holger Prokisch, Matias Wagner, Johannes A. Mayr, René G. Feichtinger, and Robert Kopajtich performed the genetic and functional diagnostics. Nicole Hammann and Lea Dewi Schlieben were responsible for statistical analysis and figure composition. Nicole Hammann, Dominic Lenz, and Christian Staufner drafted the article. All authors critically revised the content and approved the final manuscript.

## Ethics Statement

The study was approved by the ethical committee of the Technical University Munich and the ethical committee of the University Hospital Heidelberg.

## Consent

All procedures followed were in accordance with the ethical standards of the responsible committee on human experimentation (institutional and national) and with the Helsinki Declaration of 1975, as revised in 2024. Informed consent to participate in the study was obtained from all individuals or their parents in case of minors.

## Conflicts of Interest

The authors declare no conflicts of interest.

## Supporting information


Figures S1 and S2.

**Data S1.** Case Reports.

Table S1.

## Data Availability

All datasets are available from the corresponding author on reasonable request. Data will be de‐identified prior to sharing with third persons.
