# Supplementary material for: Hepatic Form of Dihydrolipoamide Dehydrogenase Deficiency (DLDD): Phenotypic Spectrum, Laboratory Findings, and Therapeutic Approaches in 52 Patients
Source: J Inherit Metab Dis. 2025 May 19;48(3):e70035. doi: 10.1002/jimd.70035 (PMC12089891; doi:10.1002/jimd.70035)
Supplement: Supplementary file 1 — Figures S1 and S2. [file JIMD-48-0-s001.docx]

**DLD-7**


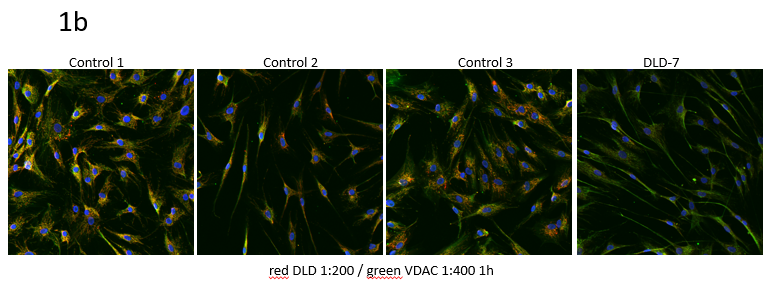


red DLD/green VDAC1

**Supplemental Figure S1:** 1A confirmatory diagnostic for DLD deficiency of individual DLD-7. A. Reduced PDH activity in DLD-7 fibroblasts. 1B immunofluorescence for DLD shows clearly reduced levels in in DLD-7 fibroblasts compared to three controls. DLD is detected with a red secondary antibody (594 nm) and VDAC1 (488 nm) with a green one. Therefore, a reduction of DLD is seen by a shift to green. PDH, pyruvate dehydrogenase; VDAC1, voltage-dependent anion channel.

| **DLD-1**  **Supplemental Figure S2:** Development of decompensations over time and upon therapeutic approaches. Age at decompensation and laboratory values for ASAT, ALAT, INR, lactate (each maximal value) and glucose (minimal value) for each decompensation are plotted over the lifespan of each affected individual (see also supplementary table S1). The introduction of each new medication or relevant change in medication is marked by an arrow. Approximate reference values are given in green/grey for comparison, though those are not age-, gender- and laboratory-adjusted.  NAC  riboflavin  thiamine | **DLD-2**  riboflavin, vitamin E, coenzyme Q10 and l-carnitine  Unknown amount of episodes | **DLD-3**  no continuous therapy | **DLD-4**  NAC, l-carnitine  bicarbonate  lipoic acid  thiamine  riboflavin, high carbohydrate diet  IVIG  PEG | **DLD-5**  riboflavin | **DLD-6**  riboflavin | **DLD-7**  NAC  Late meals and MCT oil  thiamine and riboflavin  PEG  starch  vitamin k |
| --- | --- | --- | --- | --- | --- | --- |
| **** | **** | 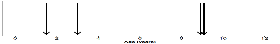 | 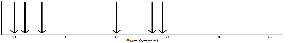 | 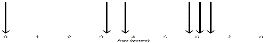 | 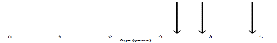 | **** |
| 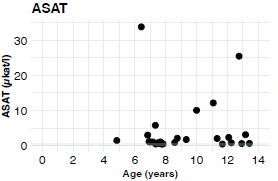 | 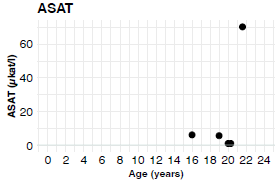 | 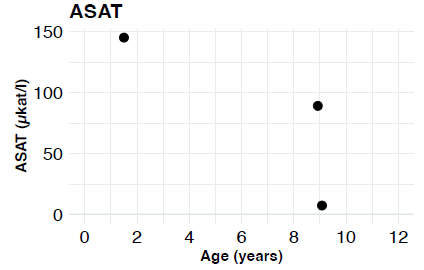 | 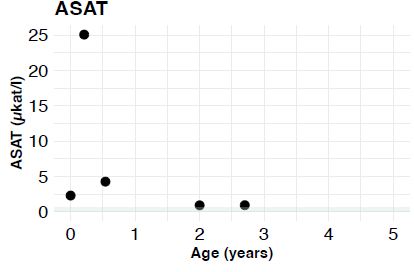 | 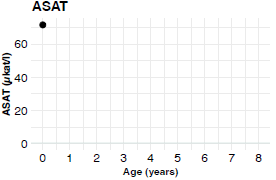 | 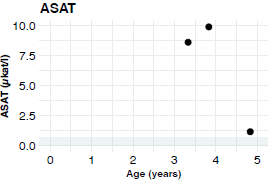 | 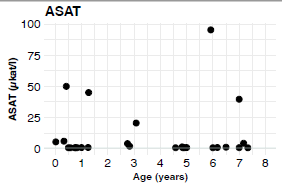 |
| 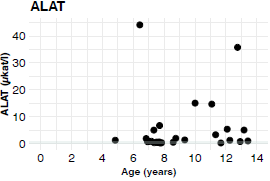 | 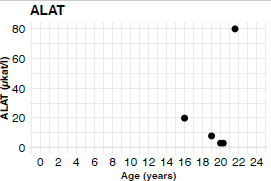 | 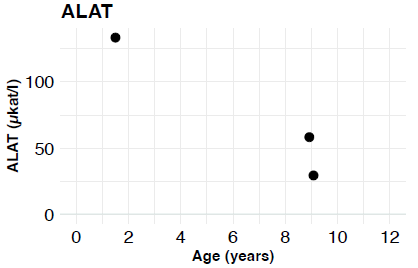 | 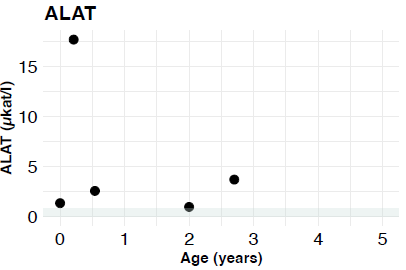 | 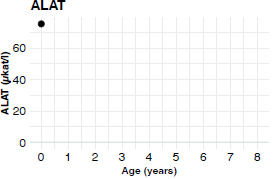 | 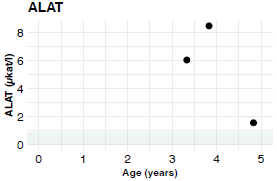 | 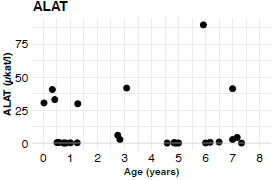 |
| 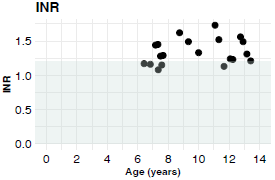 | 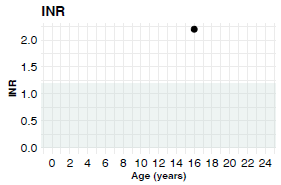 | 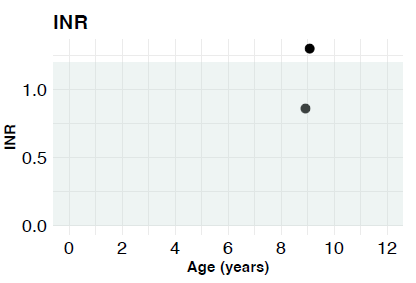 | 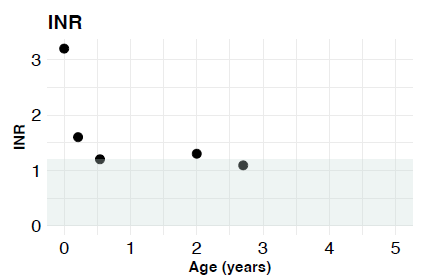 | 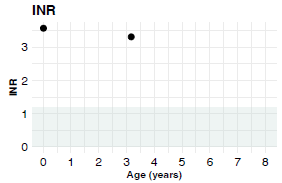 | 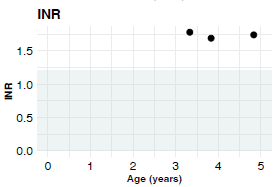 | 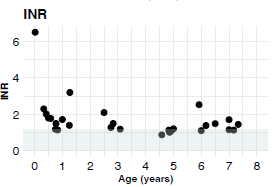 |
| 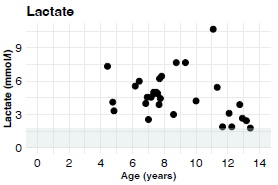 | 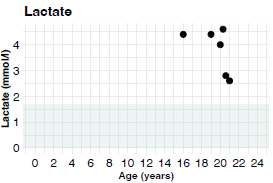 | 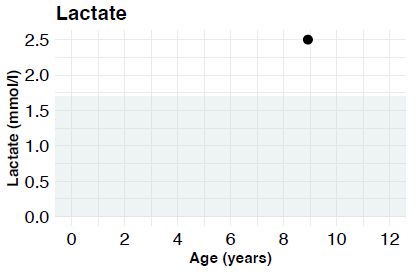 | 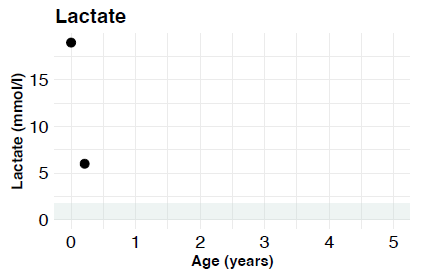 | 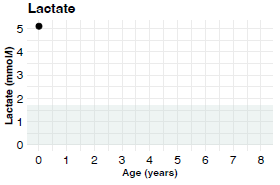 | 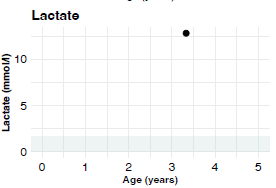 | 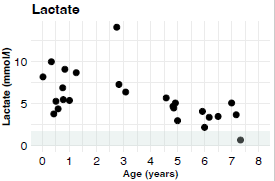 |
| 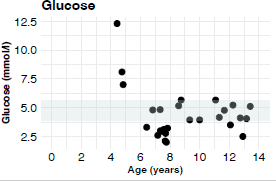 |  | 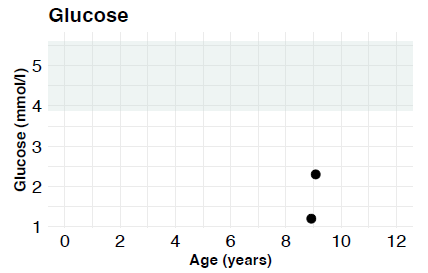 | 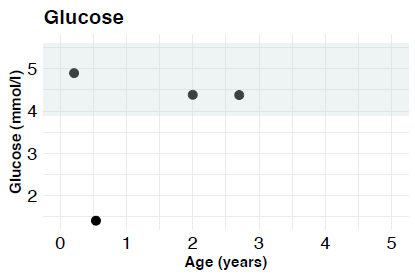 | 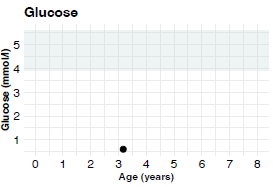 |  | 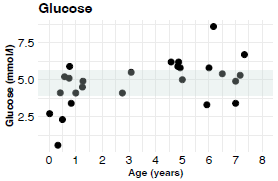 |

**Supplemental Figure S2:** Development of decompensations over time and upon therapeutic approaches. Age at decompensation and laboratory values for AST, ALT, INR, lactate (each maximal value) and glucose (minimal value) for each decompensation are plotted over the lifespan of each affected individual (see also supplementary table S1). The introduction of each new medication or relevant change in medication is marked by an arrow. Approximate reference values are given in green for comparison, though those are not age-, gender- and laboratory-adjusted.
