# Supplementary material for: Hepatic Form of Dihydrolipoamide Dehydrogenase Deficiency (DLDD): Phenotypic Spectrum, Laboratory Findings, and Therapeutic Approaches in 52 Patients
Source: J Inherit Metab Dis. 2025 May 19;48(3):e70035. doi: 10.1002/jimd.70035 (PMC12089891; doi:10.1002/jimd.70035)
Supplement: Supplementary file 2 — Data S1. Case Reports. [file JIMD-48-0-s003.docx]

**CASE REPORTS**

**DLD-1**

DLD-1, a boy, was born in Germany as the first child to consanguineous healthy Lebanese parents with 41 weeks of gestational age by spontaneous birth. Birth weight and length were normal. There were no complications during pregnancy and also neonatal period was unremarkable. Two younger siblings are healthy (aged 5 years and 11 years), a younger brother suffered from a syndromic disease with complex cardiac anomaly (hypoplastic left ventricle, hypoplastic aortic arc), microcephaly, retention testis and a sinus urogenitalis and died at the age of 18 months. There is no family history for liver disease.

Apart from a subcutaneous hemangioma on the right frontal forehead and a hospitalization due to RSV bronchiolitis at the age of 5 weeks, there were no medical issues in the first two years of life. From the 3^rd^ year of life, the boy repeatedly presented at the outpatient department due to recurrent vomiting associated with febrile infections of the upper airways, leading to dehydration. With 4.4 years of age he was first hospitalized to this clinical pattern. Lactic acidemia (5.2 mmol/L) was observed; liver function tests were not performed but a mild hepatomegaly was noted. In the following years, frequent hospitalization was necessary due to recurrent vomiting associated with lactic acidemia (lactate max. 10.7) and/or elevated liver transaminases often triggered by febrile illness. At the age of 6.4 years he had a first episode with massive hepatocytolysis (ASAT 2028 U/L max., ALAT 2647 U/L max.), albumin was slightly reduced and GGT slightly elevated, coagulation was normal. He had lactic acidemia. Workup for elevated transaminases ruled out Hepatitis A, B, C and E as EBV and CMV, furthermore autoimmune hepatitis, Alpha-1-antitrypsin-deficiency and M. Wilson. Metabolic workup including organic acids in urine, amino acids in plasma, long chain fatty acids in plasma, acylcarnitinprofile in dried blood spots, chitotriosidase activity, and isoelectric focusing of serum transferrin was without specific findings. It showed unspecific slightly increased free fatty acids and ketonemia, furthermore slightly elevated glycine and homocysteine, organic acids were unremarkable. At the age of 7.2 years via whole exome sequencing, a homozygous, pathogenic variant in *DLD* was identified (DLD NM_000108.3: c685G>T, p.[Gly229Cys].

The family was instructed to present to the outpatient department with any signs of vomiting or decreased well-being immediately for emergency treatment. Treatment with thiamine 3x100mg and subsequently riboflavin 3x50 mg was started at 7.4 and 7.8 years respectively. Acute episodes were treated with intravenous rehydration and administration of glucose, furthermore, thiamine and riboflavin were administered intravenous during hospitalizations. At the age of 11.1 years N acetyl-cysteine (NAC) was added for hepatoprotection during acute decompensations. Upon admission and emergency management episodes mostly resolved within one to three days. He was admitted up to eleven times per year (8^th^ year of life) for suspected or beginning metabolic decompensation, with increasing age and upon therapy crises started getting first milder, then less frequent with one to three admissions per year in the following years. Only during the last year (13^th^ year of life), he was admitted five times, often the trigger factor was not known, sometimes stress or incompliance to medication were documented.

Usually an episode would start with abdominal pain followed by vomiting, accompanied by lactic acidemia. Sometimes, episodes led to hypoglycemia (min 36 mg/dL). After the first, more severe episode at the age of 6.5 years, further similar episodes with prominent hepatocytolysis and beginning derangement of coagulation were reported at 6.8 years, 7.4 years, 7.6 years, 10 years, 11.1 years, 12.1 years, 12.7 years and 13.2 years. In episodes with liver involvement usually a mild elevation of transaminases was followed by an increase of INR (INR max. 1.73) and subsequently sometimes a massive increase in hepatic transaminases.

In the interval, there was complete normalization of liver transaminases, amino acids, organic acids and lactate. Liver ultrasound revealed normal texture of the organ, but a remarkable persistent hepatomegaly with borderline splenomegaly. Fibroscan showed no sign of liver fibrosis, last done at 12.5 years.

At last evaluation (aged 13 years), psychomotor development is normal, neurological examination is unremarkable and echocardiography is normal, especially no signs of cardiomyopathy. The boy visits regular school, is physically active and does not report fatigue, myalgia nor muscle weakness.

**DLD-2**

DLD-2 is a girl term born to non-consanguineous healthy parents from south-east Turkey who grew up in southern Germany. Pregnancy, birth and family history is unremarkable. She has one younger sister who is unaffected. Since the age of 3 years she was repeatedly seen with episodes of nausea, vomiting and liver dysfunction accompanied by lactate acidosis. Some of these episodes were possibly triggered by infections of the upper respiratory or the gastrointestinal tract, whereby the latter could retrospectively have been a misdiagnosis and rather represent metabolic decompensation. Since the first episodes she received supportive therapy for suspected mitochondrial disease (vitamin B2 (20 mg in two single doses; ~ 0,3 mg / kg BW), vitamin E (800 mg in two single doses; ~ 12,5 mg / kg BW), coenzyme Q10 (60 mg in a single dose; ~ 0,1 mg / kg BW) and L-carnitine (3 g in three single doses; ~ 50 mg / kg BW)) and parenteral energy supply with 2,5 - 3,5 glucose per kg body weight and day during the crisis. However, adherence to therapy was lacking in stable periods without symptoms. At the age of 16 years, she had the so far most severe decompensation most likely triggered by a gastrointestinal infection. The initial presentation in this episode was with massive bilious vomiting, increased blood lactate and total bilirubin. Acute liver failure rapidly ensued with lactate acidosis (4.4 mmol/l max.), hyperammonemia (252 µmol/l max.), coagulopathy (INR 2.2 max.), hyperbilirubinemia (2.5 mg/dl max.) and increased aminotransferases (AST 380 U/l max., ALT 1191 U/l max.). Upon clinical deterioration with hepatic encephalopathy grade 3 the patient was treated with albumin dialysis with the Molecular Adsorbent Recirculating System (MARS) unit and required artificial respiration. She fully recovered after 12 days.

Extensive laboratory and imaging tests did not reveal a specific cause for the symptoms initially. Autoimmune and viral hepatitis were excluded. A brain MRI during above mentioned crisis showed a pattern compatible with hepatic encephalopathy (decreased concentrations of choline and myoinositol; increased concentrations of glutamine and glutamate in spectroscopy; no cerebral edema). Liver ultrasound in adolescence delivered normal results, yet most recent examinations showed discrete hepatic steatosis, three liver cysts (7 - 11 mm in diameter) and borderline hepatosplenomegaly. A liver biopsy at the age of 21 years confirmed predominantly microvesicular steatosis without further abnormalities, while no histological changes were observed in an earlier liver biopsy at the age of 16 and in muscle tissue. Mitochondrial disease was excluded by analysis for pathological variants of genes relevant for liver function (e.g., *POLG, MPV17, DGUOK*) and functional analysis of enzymes in the respiratory chain pathway in muscle tissue. Other metabolic conditions, such as Wilson disease, were excluded. On one occasion a mild increase of urine organic acids (5-oxoproline, 2-oxoadipic and 2-adipic acid) was noted during an acute decompensation, but amino acid analysis had been without specific abnormalities at that time. Finally, whole exome sequencing revealed a homozygous mutation leading to p.G229C in *DLD* at the age of 24 years. After the severe episode at the age of 16 years, frequency of episodes increased until the age of 21. At last follow-up, DLD-2 was a symptom-free adult of 24 years with a normal neurological development who just graduated from university, in remission for 3 years without any medication. She neither suffered permanent fatigue nor muscle weakness and myalgia did not occur.

**DLD-3**

DLD-3, a girl, was born from third pregnancy as second child at 40 weeks gestational age with low birth weight (2560g). There was no complication during pregnancy and adaptation was normal. Family history was negative regarding liver disease.

At the age of 1.5 years she presented with fever, emesis and weakness. Liver transaminases were elevated (ASAT 8700 U/L max, ALT 8000 U/l max), whereas bilirubin and ammonia were normal and there was no coagulopathy. Laboratory work up was unremarkable regarding viral infections, autoimmune liver diseases, alfa-1-antitrypsin deficiency, and Wilson disease. Metabolic analyses including acylcarnitine profile, biotinidase activity and urinary organic acids were without specific findings. The symptoms resolved within few days and transaminases normalized within 1-2 weeks.

At the age of three years, a second decompensation occurred, where besides nausea and vomiting the girl presented somnolent and even apathic. Liver ultrasound showed hepatomegaly and increased liver echogenicity. On liver biopsy, mild chronic hepatitis and fibrosis were detected, but metabolic and storage disorders were considered unlikely. Liver biopsy was repeated at the age of 4 years and showed steatosis, mild fibrosis, minimal ductal reaction, but without signs of inflammation, liver size was normal in the interval.

Whole exome sequencing revealed the homozygous missense variant in DLD c.[685G>T], p. [Gly229Cys].

At time of report, she has experienced eight episodes of ELT usually triggered by viral infection usually starting with vomiting, some of them with mild hypoglycemia and all of them without or with only mild coagulopathy (INR max 1.21). Ammonia was always normal. Lactate and pyruvate have been measured twice in plasma in the course of two decompensations after beginning of therapy and were reported to be normal, however the lactate/pyruvate ratio was elevated up to 25.

The last episode was at the age of 9 years, starting with vomiting. She had hypoglycemia (2,3 mmol/l), hepatomegaly, ELT (AST 5345 U/L, ALR 3502 U/l) and furthermore acute pancreatitis (lipase 572 U/L, amylase 230 U/L). Ammonia, lactate and pyruvate were normal. She received intravenous glucose and fluids for four days, all laboratory abnormalities resolved within 2 weeks.

At last clinical examination at the age of twelve years, psychomotor development is normal, no neurological symptoms have been reported, she has no muscular hypotonia or exercise intolerance and attends regular school with good results. Echocardiogram was normal. Abdominal ultrasound at the age of twelve years showed normal liver size with inhomogeneous structure and “map-like” hyperreflectivity.

**DLD-4**

DLD-4 is a girl who was born as second child to non-consanguine Ashkenazi Jews after an inconspicuous pregnancy and spontaneous delivery in pregnancy week 40 + 5 with normal birth weight 3250 g (15.P.) and height 50cm (10.P.) and good postnatal adaption (APGAR 9/10/10).

The patient was presented at the age of two days with shrill crying and recurrent vomiting, hyperextension tendencies alternately with muscular hypotonia and tachydyspnea. She was transferred to the neonatal intensive care unit with severe lactic acidosis (pH 7.15, pCO2 20 mmHg, BE -20 mmol/l, lactate 19 mmol/l). Laboratory workup revealed a discrete hyperammonemia (ammonia 102.7 µmol/l (ref. 11.2-48.2), slightly elevated hepatic transaminases (ALT 137 U/l (ref. 25-85), ALT 81 U/l (ref. 12-93)), coagulopathy (thromboplastin time 15 % (ref. 70-125), aPTT 62.9 s (ref. 30-45)) and elevated CK (1741 U/l (ref. <25). Protein intake was stopped for 12 hours, she received high dose intravenous glucose (15 g/kg/d); furthermore insulin, bicarbonate, FFP, vitamin K and AT III. Upon emergency treatment a significant decrease of lactate and ammonia was reached. She had no elevated inflammatory parameters and no viral cause could be identified. Thoracic x-ray, echocardiogram, abdominal ultrasound, EEG and ophthalmologic examination were all normal, cranial ultrasound showed an increased echogenicity of the basal ganglia but cranial MRI showed no abnormalities besides a small lactate peak in the spectroscopy. After stabilization and normalization of all laboratory parameters the patient could be discharged from our clinic.

Readmission at the age of 2.5 months with vomiting and a first onset of ALF (AST max 1503 U/l, ALT max 1060 U/L, ammonia 72 µmol/l, lactate 6 mmol/l, aPTT 50s, PT 53 %, ATIII 42 %) triggered by febrile infection. At the age of 6,5 months she was readmitted due to recurrent vomiting triggered by vaccination the day before. She had severe hypoglycemia (1.4 mmol/l), also moderate metabolic acidosis and discretely elevated transaminases (pH 7.33, BE -12.4, SBIC 15.2, AST 154 U/l, ALT 254 U/l, GGT 255 U/l).

Metabolic work-up showed elevations of alanine and proline in plasma and high excretion of lactate and ketoacids in urine; furthermore, small amounts of 2-hydroxyglutaric acid and glutaric acid, and 3-methylglutaconic acid and 3-methylglutaric acid were detected, hinting at a mitochondrial disorder. After stabilization all laboratory abnormalities were normalized. Acylcarnitine profiles in were repeatedly unremarkable.

Due to the symptom constellation an inherited error of metabolism leading to recurrent febrile ALF was suspected. Eventually a repeated work-up and WES revealed compound heterozygous variants c.[1046+5G>T); p.[Ile293_Asn349del] and c.[685G>T]; p.[Gly229Cys] in *DLD* gene.

In the following the patient required hospitalizations frequently (weekly to monthly) for lactic acidosis and hypoglycemia, usually starting with vomiting. During febrile illness she usually developed ELT but mostly without ALF. Other trigger factors included vaccinations. She is treated with thiamine (since the age of 9 months), riboflavin (100 mg/d; since the third day of life) and high carbohydrate diet; in the course a PEG was implemented to ensure regular meals. At the age of 18 months, lipoic acid was added to therapy, at the age of 2.7 years NAC and levocarnitine and at 3.5 years natrium-bicarbonate. To avoid further metabolic derangement in case of fever and/or vomiting, presentation at the emergency room and application of emergency treatment with high caloric infusions was recommended. For decreasing risk of infections, she received IVIG monthly until the age of 3.3 years. At last follow-up in our facility at the age of 4.5 years she shows an age-related psychomotor and neurological development and she visits kindergarten. Echocardiogram and EKG were normal.

**DLD-5**

DLD-5 is the second child of healthy Jewish parents with no known consanguinity; one older brother and one younger sister were both considered healthy. After an uneventful pregnancy, she was born after 39 4/7 weeks by Caesarean section due to pathological CTG. Birth weight was normal (3290 g), Apgar 9/9/9. On day 3, she started to show neurological signs with muscular hypotonia and poor feeding. On day 6, she was transferred to the intensive care unit (ICU) where signs of a metabolic decompensation were found: blood gases showed metabolic acidosis (pH 7.34, pO2 6.24 kPa, pCO2 3.45 kPa, HCO3 16.7 mmol/L, BE -10.6 mmol/L, anion gap 13.3), elevated lactate (5.1 mmol/l), hyperammonaemia (333 µmol/L, ref < 150), blood glucose was normal. In addition, signs of liver failure were present (alanine aminotransferase 4299 U/L, ref < 28; aspartate aminotransferase 4523 U/L, ref < 35; INR 3.57, ref < 1.4). Plasma amino acids showed elevation of citrulline (466 µmol/L, ref 5-33) with normal branched chain amino acids. The patient was treated for a few days with glucose infusions (10 mg/kg/min), lipids (1 g/kg/d), and sodium benzoate (250 mg/kg/d), and made a fast recovery. Mutation analysis of the *ASS1* gene was normal, hereby likely excluding classical citrullinemia. During infancy, she had an episode of vomiting and was hospitalized for 2 days receiving a glucose infusion as above. Development was normal at 1 and 3 years of age. Shortly after her 3^rd^ birthday, she experienced another episode of vomiting during a non-febrile upper airway infection, but recovered fast with continued oral feeding. At 3 years 2 months, she presented with another episode of repetitive vomiting, and went on to develop prolonged status epilepticus > 45 minutes likely secondary to hypoglycaemia (0.6 mmol/L, ref 3.7-5.6), warranting intubation. Work up revealed acute liver failure (INR 3.31; factor V 11.4%, ref > 70; factor VII 3.2%, ref > 60; ammonia 100 µmol/L, ref < 48), a non-focal EEG pattern compatible with mild hepatic encephalopathy, and a Picorna virus upper airway infection. Plasma amino acids demonstrated elevation of citrulline (352 µmol/L) with normal branched chain amino acids. Urine organic acids and amino acids showed elevated metabolites of the 2-oxoacid dehydrogenases, except for the branched-chain ketoacid dehydrogenase. She benefited from supportive treatment based on a high glucose infusion rate. She was promptly extubated, remained hemodynamically stable, and liver function normalized within 48 hours. Based on the recurrence of the metabolic decompensations and the combination of her biochemical findings, the diagnosis of a DLD defect was considered and later confirmed by mutation analysis. Since the diagnosis was made, there were several further episodes at various degrees of severity with metabolic decompensations and acute liver failure. Since intervention was started immediately after onset of first symptoms, most of these episodes were of short duration and the patient usually recovered within 1 or 2 days. Given the frequency of the decompensations, treatment with riboflavin (2 x 100 mg/day p.o.) was initiated at the age of 6 years and 8 months and well tolerated by the patient. Since start of this, there were almost no further episodes in the subsequent 15 months possibly indicating towards a positive treatment effect. Currently, at the age of 7 years and 11 months she shows normal neurodevelopment, she had no further seizures and has no hepatomegaly upon clinical examination.

**DLD-6**

DLD-6 is the younger sister of DLD-5, who was considered healthy at the time of the diagnosis of DLD-5. She had her first episode of acute liver failure during a minor viral illness at age 3y4m. Based on the family history, the diagnosis was immediately confirmed by genetic analysis of the *DLD* gene showing homozygosity for the same known mutation. Since then, she is treated by the same measures as her older sister, mainly receiving immediate medical management at first symptoms. As her sister she shows a normal neurodevelopment at time of report at 4.8 years.

**DLD 7**

DLD 7 is a female born at 40 weeks gestational age with normal birth weight after an uneventful pregnancy as first child to non-consanguineous parents of German origin. There is no history of liver disease in the family. At day seven she was seen at the hospital for elevated leucine/isoleucine in the newborn screening (458 µmol/l; ref. <300 µmol/l), clinically she was doing well. The next day though she presented with hyperexcitability and had two generalized tonic-clonic seizures in the following, laboratory findings included severe lactic acidosis and acute liver failure with elevated transaminases, coagulopathy and hyperammonemia; MRI of cerebrum and abdomen were unremarkable. She recovered within 9 days after supportive therapy. Similar episodes with different combinations of liver failure or elevated hepatic transaminases, hypoglycemia and lactic acidemia, usually beginning with recurrent vomiting and/or abdominal pain occurred recurrently. Episodes were often triggered by febrile illness, often gastroenteritis was suspected (although this might have been misinterpretation, often vomiting was the only symptom); fasting situations were also suspected (alternatively reduced appetite as first symptom) and also the second meningococcus b led to a crisis. Sometimes the trigger remained unclear. A liver biopsy was done at the age of 7 months immediately following an acute crisis, it showed mild swelling of hepatocytes, middle sized lipid droplets and minimal portal inflammation, also mild periportal copper accumulation was evident.

While she had many crises resulting in hospitalization in the first year of life (eight episodes); episodes became less frequent from the 2^nd^ year of life. Because of recurrent coagulopathy (also independent from ALF), she received twice daily peroral vitamin k since the age of 4 months, this was discontinued at the age of 17 months and coagulation was normal afterwards with exception of acute crises. Fasting episodes and even more so during infections led to recurrent hypoglycemia, thus, blood glucose level was monitored and oral starch application was initiated at the age of 7 months. In the further course at the age of 1.3 years, a PEG was implanted to ensure regular meals. She had often recurrent infections, apart from slightly reduced IgG no abnormalities of the immune system were found. She did not visit kindergarten until the age of five years for fear of further infections.

At the age of 3.1 years (01/2020) via whole exome sequencing, a heterozygous pathogenic variant in *DLD* was identified (DLD NM_000108.3: c685G>T, p.[Gly229Cys]), via RNA-sequencing a monoallelic expression of the pathogenic variant was proven (see supplemental figure 1). A specific therapy with thiamine 2 x 100 mg and riboflavin 2 x 50 mg was started orally. For about 1.5 years hereafter she developed no further episodes despite recurrent infections and blood sugar was more stable, even without continuous feeding overnight. Examination at the age of 3.7 years revealed persistent hepatomegaly with a normal fibroscan. She had mild development delay of speech, which might be either due to the lack of social contacts and many hospital admissions or due to the DLD deficiency itself. She started logopedia. Motoric development was normal at 3.7 years.

Starting at the age of 4.7 years she again suffered recurrent episodes starting with vomiting and decreased well-being with lactic acidosis, hypoglycemia and hepatocytolysis. As those episodes often started at night-time without infections triggering them and the patient had fasting-times of up to 15 hours over night (since the PEG was removed at the age of 3.3 years), late meals and MCT oils were added to the therapy. With avoiding of fasting situations crises started being less frequent again. At 4.8 years, NAC was added to the therapy for hepatoprotection. At the age of 5.9 years she developed a severe metabolic decompensation due to an influenza infection with elevation of hepatic transaminases and admission to the intensive care unit. Since then she had only mild and infrequent crises, however recurrent abdominal pain and hypoglycemia after exercise and during infections. Because of continuous discomfort and infrequent severe crises, the beginning of a ketogenic diet was discussed multiple times with the family, initiation is planned for this year.

In between the crises she started complaining more and more about muscle pains, exertional fatigue and abnormally much need for sleep since age 4. Currently, at the age of 7.3 years she has some mild persistent development delay of speech, she goes to school kindergarten with a medical aid for monitoring of hypoglycemia. Her last ultrasound and fibroscan at the age of 7.1 years revealed no abnormalities apart from slight hepatomegaly and minimal free fluid which might be physiological. Echocardiogram was repeatedly normal.
