# Supplementary material for: Hepatic Form of Dihydrolipoamide Dehydrogenase Deficiency (DLDD): Phenotypic Spectrum, Laboratory Findings, and Therapeutic Approaches in 52 Patients
Source: J Inherit Metab Dis. 2025 May 19;48(3):e70035. doi: 10.1002/jimd.70035 (PMC12089891; doi:10.1002/jimd.70035)
Supplement: Supplementary file 3 — Table S1. [file JIMD-48-0-s002.docx]

**Supplemental Table S1:** Genetic and phenotypic characterization and therapeutic approach of previously reported individuals with hepatic DLD deficiency.

|  | **Elpeleg et al., 1995; Elpeleg et al., 1997; Shaag et al., 1999** | **Elpeleg et al., 1997; Shaag et al., 1999** | **Aptowitzer et al., 1997; Shaag et al., 1999** | **Shaag et al., 1999; Elpeleg et al., 1990** | | | | | **Shaag et al., 1999** | | | **Shaag et al., 1999, Elpeleg et al., 1997** | **Shaag et al., 1999** |
| --- | --- | --- | --- | --- | --- | --- | --- | --- | --- | --- | --- | --- | --- |
| **ID** | Patient 2/ F1; II-3 | Patient 1/ F2; II-3 | F4; II-4 | F3; II-2 | F3; II-5 | F3; II-6 | F3; II-7 | F3; II-12 | F4; II-3 | F5; II-1 | F6; II-3 | F7; II-3 | F7; II-4 |
| **sex** | M | M | M | F | M | F | M | F | M | F | M | M | F |
| **nationality** | Israeli | Israeli | Israeli | Israeli | Israeli | Israeli | Israeli | Israeli | Israeli | Israeli | Russian | Israeli | Israeli |
| **descent** | Ashkenazi-Jewish | Ashkenazi-Jewish | Ashkenazi-Jewish | Ashkenazi-Jewish | Ashkenazi-Jewish | Ashkenazi-Jewish | Ashkenazi-Jewish | Ashkenazi-Jewish | Ashkenazi-Jewish | Ashkenazi-Jewish | Ashkenazi-Jewish | Ashkenazi-Jewish | Ashkenazi-Jewish |
| **age at last visit/report** | 8 ys | 5 ys | 3 ys | 30 ys | 13-30 ys | 13-30 ys | 13-30 ys | 13 ys | 5 ys | 19 ys | 5 ys | 37 ys | 34 ys |
| **alive at time of report?**  **If no, cause of death** | Y | Y | N, sepsis after muscle biopsy | Y | Y | Y | Y | Y | Y | Y | Y | N, metabolic decompensation | Y |
| ***DLD* variant, allele 1** | c.[685G>T]; p.[Gly229Cys] | c.[685G>T]; p.[Gly229Cys] | c.[685G>T]; p.[Gly229Cys] | c.[685G>T]; p.[Gly229Cys] | c.[685G>T]; p.[Gly229Cys] | c.[685G>T]; p.[Gly229Cys] | c.[685G>T]; p.[Gly229Cys] | c.[685G>T]; p.[Gly229Cys] | c.[685G>T]; p.[Gly229Cys] | c.[685G>T]; p.[Gly229Cys] | c.[685G>T]; p.[Gly229Cys] | c.[685G>T]; p.[Gly229Cys] | c.[685G>T]; p.[Gly229Cys] |
| ***DLD* variant, allele 2** | c.[105insA]; p.[Tyr35Xaa] | c.[105insA]; p.[Tyr35Xaa] | c.[685G>T]; p.[Gly229Cys] | c.[685G>T]; p.[Gly229Cys] | c.[685G>T]; p.[Gly229Cys] | c.[685G>T]; p.[Gly229Cys] | c.[685G>T]; p.[Gly229Cys] | c.[685G>T]; p.[Gly229Cys] | c.[685G>T]; p.[Gly229Cys] | c.[685G>T]; p.[Gly229Cys] | c.[685G>T]; p.[Gly229Cys] | c.[685G>T]; p.[Gly229Cys] | c.[685G>T]; p.[Gly229Cys] |
| **characterization of decompensations** | | | | | | | | | | | | | |
| **recurrent decompensations** | Y, every 6-8 weeks | Y | Y | Y | Y | Y | Y | Y |  |  | Y | Y |  |
| **age at first decompensation** | 12 h | 2 d | 2 ys | 2-3 ys | 2-3 ys | 2-3 ys | 2-3 ys | 2-3 ys | 1-2 ys | 3 ys | 3 ys | 20 ys | 34 ys |
| **age at most severe decompensation** |  |  |  |  | 16 ys |  |  |  |  |  |  | 37 ys |  |
| **age at last decompensation** |  |  |  | 13 ys |  |  |  |  |  |  |  | 37 ys |  |
| **ELT (HP:0002910)** | Y | Y | Y | Y | Y | Y | Y | Y | Y | Y | Y | Y | Y |
| **ALF (HP:0006554)** |  |  | Y |  |  | Y |  |  |  |  |  |  |  |
| **nausea and vomiting (HP:0002017)** | Y | Y | Y | Y | Y | Y | Y | Y | Y | Y | Y | Y | Y |
| **hepatomegaly (HP:0002240)** | Y | Y | Y | Y | Y | Y | Y | Y | Y | Y | Y | Y | Y |
| **hypoglycemia (HP:0001943)** |  |  |  | Y | Y | Y | Y | Y |  |  |  |  |  |
| **lactic acidosis (HP:0003128)** | Y | Y | Y | Y | Y | Y | Y | Y | Y | Y | Y | Y | Y |
| **triggered by febrile illness (HP:0025215)** |  | Y | Y |  |  |  |  |  |  |  | Y |  | Y |
| **triggered by fasting (HP:0025212)** |  |  |  |  |  |  |  |  |  |  |  |  |  |
| **others symptoms during decompensation** | loss of appetite, tachypnea, tachycardia, muscular hypotonia, moderate neurological deterioration, lethargy, abdominal pain | tachypnea, lethargy, abdominal pain | depressed consciousness, abdominal pain | abdominal pain | abdominal pain, hepatic encephalopathy with coma and brain edema | abdominal pain, malaise, hepatic encephalopathy | abdominal pain | abdominal pain | abdominal pain | abdominal pain | abdominal pain | abdominal pain, muscle weakness, symmetric generalized hyporeflexia, rhabdomyolysis (CK max. 71000 U/L), encephalopathy | abdominal pain |
| **Other triggering factors** |  |  |  |  |  |  |  |  |  |  |  |  |  |
| **extrahepatic symptoms** | | | | | | | | | | | | | |
| **cognitive impairment (HP:0100543)** | N | N | N | N | N | N | N | N | N | N | N | N |  |
| **muscular hypotonia (HP:0001252)** | Y | Y | N | N | N | N | N | N | N | N | N | N | N |
| **post-exertional symptom exacerbation (HP:0030973)** |  |  |  | Y | Y | Y | Y | Y |  | Y |  | Y |  |
| **others** | motor development moderately impaired, ADD, mild ataxia, motor incoordination, impaired vision (suspected cortical damage) | motor incoordination, ADHD, mild ataxia |  |  | bilateral impaired vision and moderate behavioral disturbances after severe episode at 16 years |  |  |  |  |  | suspected vit B1 deficiency upon DCA |  |  |
| **therapy** | | | | | | | | | | | | | |
| **B1 (thiamine)** | Y | Y | Y |  |  |  |  |  |  |  | Y |  |  |
| **B2 (riboflavin)** | Y | N | N |  |  |  |  |  |  |  | N |  |  |
| **NAC** | N | N | N |  |  |  |  |  |  |  | N |  |  |
| **ketogenic diet** | N | N | N |  |  |  |  |  |  |  | N |  |  |
| **carnitine** | Y | Y | Y |  |  |  |  |  |  |  | N |  |  |
| **lipoic acid** | Y | N | N |  |  |  |  |  |  |  | N |  |  |
| **MCT** | N | N | N |  |  |  |  |  |  |  | N |  |  |
| **others** | DCA, coenzyme Q, biotin, NA succinate, restriction branched-chain amino acid | DCA | DCA |  |  |  |  |  |  |  | DCA and frequent feeding |  |  |
| **improvement seen on:** | DCA, thiamine and carnitine | DCA, thiamine and carnitine |  |  |  |  |  |  |  |  | thiamine |  |  |

|  | **Hong et al., 2003** | | **Sansaricq et al., 2006** | **Brassier et al., 2013** | | | **Cameron et al., 2006** | **Hegarty et al., 2019** | **Neveu et al., 2020** |
| --- | --- | --- | --- | --- | --- | --- | --- | --- | --- |
| **ID** | Patient 2 | Patient 4 |  | Patient 3 | Patient 4 | Patient 5 | Patient 1 | Patient 45 |  |
| **sex** | M | F | M | M | M | M | M | M | M |
| **nationality** | Palestinian |  | US-American | Algerian | Algerian | Algerian | Canadian | British | French |
| **descent** | Arab | Ashkenazi-Jewish | Ashkenazi-Jewish |  |  |  | Ashkenazi-Jewish |  |  |
| **age at last visit/report** | 16 ys | 5 ys | 4 ys | 39 ys | 31 ys | 21 ys | 8.5 ys | 17 ys | 11 ys |
| **alive at time of report?**  **If no, cause of death** | Y | N, deceased with hepatic failure | Y | Y | Y | Y | Y | Y | Y |
| ***DLD* variant, allele 1** | c.[685G>T]; p.[Gly229Cys] | c.[685G>T]; p.[Gly229Cys] | c.[685G>T]; p.[Gly229Cys] | c.[685G>T]; p.[Gly229Cys] | c.[685G>T]; p.[Gly229Cys] | c.[685G>T]; p.[Gly229Cys] | c.[685G>T]; p.[Gly229Cys] | c.[685G>T]; p.[Gly229Cys] | c.[685G>T]; p.[Gly229Cys] |
| ***DLD* variant, allele 2** | c.[685G>T]; p.[Gly229Cys] | c.[685G>T]; p.[Gly229Cys] | c.[685G>T]; p.[Gly229Cys] | c.[685G>T]; p.[Gly229Cys] | c.[685G>T]; p.[Gly229Cys] | c.[685G>T]; p.[Gly229Cys] | c.[?];  p.[Ile47Thr] | c.[685G>T]; p.[Gly229Cys] | c.[685G>T]; p.[Gly229Cys] |
| **characterization of decompensations** | | | | | | | | | |
| **recurrent decompensations** | Y | Y | Y | Y | Y | Y | Y | Y | Y |
| **age at first decompensation** | 8 m |  | 8 m | 4 ys |  | 0 d | 2 d | 9 ys | 5 ys |
| **age at most severe decompensation** |  | 5 ys | 4 ys |  |  | 14 ys |  |  |  |
| **age at last decompensation** |  | 5 ys |  | 38 ys | 30 ys | 21 ys | 8.5 ys |  |  |
| **ELT (HP:0002910)** |  |  | Y | Y | Y | Y | Y | Y | Y |
| **ALF (HP:0006554)** |  | Y |  | Y | Y | Y | Y | Y | Y |
| **nausea and vomiting (HP:0002017)** | Y | Y | Y | Y | Y | Y |  |  |  |
| **hepatomegaly (HP:0002240)** | Y |  | Y | N |  |  |  |  |  |
| **hypoglycemia (HP:0001943)** |  |  | Y | Y |  | Y | N |  | Y |
| **lactic acidosis (HP:0003128)** | Y |  | Y | Y |  | Y | Y |  | Y |
| **triggered by febrile illness (HP:0025215)** |  |  | Y | Y | Y | Y | Y |  |  |
| **triggered by fasting (HP:0025212)** |  |  |  | Y | Y | Y | Y |  | Y |
| **others symptoms during decompensation** | encephalopathy, somnolence, general muscle weakness, wasting, ataxic gait, pyramidal signs |  | apathy, hypotonia, seizures, loss of deep tendon reflexes, | anorexia; somnolence, cramps, muscle weakness |  | acute gastric hemorrhage, encephalopathy | seizure, myocardial dysfunction, MRI during ALF: abnormal signal in the basal ganglia and the frontal lobes |  | acute encephalopathy |
| **Other triggering factors** |  |  |  | triggered by excessive meals or tiredness | triggered by tiredness | triggered by fatty food |  |  |  |
| **extrahepatic symptoms** | | | | | | | | | |
| **cognitive impairment (HP:0100543)** | N |  | N | N | N | N | N |  |  |
| **muscular hypotonia (HP:0001252)** | Y |  | Y | N | N | N | N |  |  |
| **post-exertional symptom exacerbation (HP:0030973)** |  |  |  | Y | Y | N |  |  |  |
| **others** | ataxia |  | Tonic-clonic seizures, absent deep tendon reflexes | muscle cramps | Feels better when drinking high sugar sodas, muscle cramps |  |  |  |  |
| **therapy** | | | | | | | | | |
| **B1 (thiamine)** | N | N | N |  |  |  |  |  | N |
| **B2 (riboflavin)** | Y | N | N | did not help | did not help | did not help |  |  | Y |
| **NAC** | N | N | N |  |  | yes, in acute episode |  |  | N |
| **ketogenic diet** | N | N | N |  |  |  |  |  | N |
| **carnitine** | Y | N | N |  |  | yes, in acute episode |  |  | Y |
| **lipoic acid** | N | N | Y |  |  |  |  |  | N |
| **MCT** | N | n | N |  |  |  |  |  | N |
| **others** | coenzyme Q, biotin |  |  |  |  |  |  |  | limit protein intake during illness |
| **improvement seen on:** | all above |  |  |  |  |  |  |  |  |

|  | **Alfarsi et al., 2021** | | | | **Siri et al., 2022** | | **Ramadža et al., 2021/ Mihaljevic et al., 2024** | **Wongkittichote et al., 2023** | | | **Moosavian et al., 2024** |
| --- | --- | --- | --- | --- | --- | --- | --- | --- | --- | --- | --- |
| **ID** | Patient 1 | Patient 2 | Patient 3 | Patient 4 | Subject 10 | Brother of Subject 10 | Patient 1 | Patient 1 | Patient 3 | Patient 4 |  |
| **sex** | M | F | M | F | F | M | M | F | F | F | M |
| **nationality** | Saudi Arabian | Saudi Arabian | Saudi Arabian | Saudi Arabian |  |  | Croatian | US-American | US-American | US-American | Iranian |
| **descent** |  |  |  |  | Roma | Roma | Roma | Ashkenazi-Jewish | Ashkenazi-Jewish | Ashkenazi-Jewish |  |
| **age at last visit/report** |  |  |  |  | 15 ys | 17 ys | 7 ys |  |  |  | 16 ys |
| **alive at time of report?**  **If no, cause of death** | Y | Y | Y | Y | Y | Y | N, death due to sepsis as complication from multiorgan failure due to acute decompensation and liver transplantation | Y | Y | Y | y |
| ***DLD* variant, allele 1** | c.[685G>T]; p.[Gly229Cys] | c.[685G>T]; p.[Gly229Cys] | c.[685G>T]; p.[Gly229Cys] | c.[685G>T]; p.[Gly229Cys] | c.[685G>T]; p.[Gly229Cys] | c.[685G>T]; p.[Gly229Cys] | c.[685G>T];  p.[Gly229Cys] | c.[685G>T]; p.[Gly229Cys] | c.[685G>T]; p.[Gly229Cys] | c.[685G>T]; p.[Gly229Cys] | c.[685G>T];  p.[Gly229Cys] |
| ***DLD* variant, allele 2** | c.[685G>T]; p.[Gly229Cys] | c.[685G>T]; p.[Gly229Cys] | c.[685G>T]; p.[Gly229Cys] | c.[685G>T]; p.[Gly229Cys] | c.[685G>T]; p.[Gly229Cys] | c.[685G>T]; p.[Gly229Cys] | c.[685G>T];  p.[Gly229Cys] | c.[685G>T]; p.[Gly229Cys] | c.[1046+5G>T];  p.[?] | c.[685G>T]; p.[Gly229Cys] | c.[685G>T];  p.[Gly229Cys] |
| **characterization of decompensations** | | | | | | | | | | | |
| **recurrent decompensations** | Y | Y | Y |  | Y | Y | N | Y | Y | Y | Y |
| **age at first decompensation** | 2 ys | 16 ys | 3 ys | 5 m |  |  | 7 ys |  |  |  | 2 ys |
| **age at most severe decompensation** |  |  |  |  |  |  | 7 ys |  |  |  | 16 ys |
| **age at last decompensation** |  |  |  |  |  |  | 7 ys |  |  |  | 16 ys |
| **ELT (HP:0002910)** | Y | Y | Y | Y | Y | Y | Y | Y | Y | Y | Y |
| **ALF (HP:0006554)** | N |  | Y |  |  | Y | Y | Y | Y | Y | Y |
| **nausea and vomiting (HP:0002017)** |  | Y |  |  |  |  | Y |  |  |  | Y |
| **hepatomegaly (HP:0002240)** |  |  |  |  |  |  | Y |  |  |  |  |
| **hypoglycemia (HP:0001943)** | Y | N | N | N |  |  | N |  | Y |  |  |
| **lactic acidosis (HP:0003128)** | Y | Y | Y | N |  | Y | Y | Y | Y | Y | Y |
| **triggered by febrile illness (HP:0025215)** |  |  |  |  |  |  | Y |  |  |  | Y |
| **triggered by fasting (HP:0025212)** | Y |  |  |  |  |  | N |  |  |  | N |
| **others symptoms during decompensation** | encephalopathy | encephalopathy | encephalopathy |  | Recurrent abdominal pain | Recurrent abdominal pain, hyperammonemic encephalopathy, selective protein aversion | Abdominal pain, encephalopathy, cardiac arrest and multi-organ failure |  |  |  | behavioral changes, decreased consciousness, rhabdomyolysis, progressive hearing loss, vision impairment, irregular eye movement, weakness lower limb muscles, diminished deep tendon reflexes |
| **Other triggering factors** |  |  |  |  |  |  |  |  |  |  |  |
| **extrahepatic symptoms** | | | | | | | | | | | |
| **cognitive impairment (HP:0100543)** | N | N | N | Y | N | N | N | N | N | N | N |
| **muscular hypotonia (HP:0001252)** | N | N | N | N | N | N | N |  |  |  | N |
| **post-exertional symptom exacerbation (HP:0030973)** |  |  |  |  | Y | N |  |  |  |  |  |
| **others** |  | hypertrophic cardiomyopathy |  | seizures, microcephaly; cMRI: symmetrical increased T2-weighted signal intensity affecting putamen and caudate nuclei with atrophic changes |  |  | bilateral thalamic lesions |  |  |  |  |
| **therapy** | | | | | | | | | | | |
| **B1 (thiamine)** | Y | Y | Y | N | Y | Y | N |  |  |  | Y |
| **B2 (riboflavin)** | Y | Y | Y | N | Y | Y | N |  |  |  | discussed |
| **NAC** | N | N | N | N | N | N | N |  |  |  | N |
| **ketogenic diet** | N | N | N | N | N | N | N |  |  |  | N |
| **carnitine** | Y | Y | Y | N | N | N | N |  |  |  | N |
| **lipoic acid** | N | N | N | N | N | N | N |  |  |  | N |
| **MCT** | N | N | N | N | N | N | N |  |  |  | N |
| **others** | coenzyme Q10, biotin | coenzyme Q10, biotin | coenzyme Q10, biotin | levetiracetam |  |  | liver transplantation |  |  |  | plasmapheresis, not effective |
| **improvement seen on:** |  |  | all above (no episodes for 3 years after therapy start) |  |  |  | / |  |  |  |  |

|  | **Pode-Shakked et al., 2024** | | | | | | | | | | | |
| --- | --- | --- | --- | --- | --- | --- | --- | --- | --- | --- | --- | --- |
| **ID** | 3 | 4 | 10 | 17 | 37 | 46 | 47 | 48 | 49 | 50 | 51 | 52 |
| **sex** | F | M | F | F | F | M | M | F | F | F | M | F |
| **nationality** | Israeli | Israeli | Israeli | Israeli | Israeli | Israeli | Israeli | Israeli | Israeli | Israeli | Israeli | Israeli |
| **descent** | Ashkenazi-Jewish | Ashkenazi-Jewish | Ashkenazi-Jewish | Ashkenazi-Jewish | Bedouin | Ashkenazi/Libya-Jewish | Ashkenazi-Jewish | Ashkenazi-Jewish | Ashkenazi-Jewish | Ashkenazi-/Sephardic-Jewish | Ashkenazi-Jewish | Ashkenazi-Jewish |
| **age at last visit/report** | 4 ys | 7 ys | 6.5 ys | 47.7 ys | 3 m | 9.5 ys | 16.5 ys | 11 ys | 6 ys | 1 ys | 30 ys | 3.5 ys |
| **alive at time of report?**  **If no, cause of death** | Y | Y | N | N, death due to metabolic decompensation | N | Y | Y | Y | Y | Y | Y | Y |
| ***DLD* variant, allele 1** | c.[685G>T]; p.[Gly229Cys] | c.[685G>T]; p.[Gly229Cys] | c.[685G>T]; p.[Gly229Cys] | c.[685G>T]; p.[Gly229Cys] | p.D479V | c.[685G>T]; p.[Gly229Cys] | c.[685G>T]; p.[Gly229Cys] | c.[685G>T]; p.[Gly229Cys] | c.[685G>T]; p.[Gly229Cys] | c.[685G>T]; p.[Gly229Cys] | c.[685G>T]; p.[Gly229Cys] | c.[685G>T]; p.[Gly229Cys] |
| ***DLD* variant, allele 2** | c.[685G>T]; p.[Gly229Cys] | c.[685G>T]; p.[Gly229Cys] | c.[685G>T]; p.[Gly229Cys] | c.[685G>T]; p.[Gly229Cys] | p.D479V | p.[G53E] | p.[Y35X] | p.[Y35X] | p.[Y35X] | c.[363_367delCAAGA] | p.[Y35X] | c.[685G>T]; p.[Gly229Cys] |
| **characterization of decompensations** | | | | | | | | | | | | |
| **recurrent decompensations** |  |  | Y |  |  |  |  |  |  |  |  |  |
| **age at first decompensation** | 3 m | 6 ys | 1 ys | 6 ys | 2w | 14 h | 3 d |  | 3 d | 3 d |  | 21 m |
| **age at most severe decompensation** |  |  | 6.5 ys | 47.7 ys | 3 m |  |  |  |  |  |  |  |
| **age at last decompensation** |  |  | 6.5 ys | 47.7 ys | 3 m |  |  |  |  |  |  |  |
| **ELT (HP:0002910)** | Y | Y | Y | Y | Y | Y | Y | Y | Y | Y | Y | Y |
| **ALF (HP:0006554)** |  |  |  |  |  |  |  |  |  | Y |  |  |
| **nausea and vomiting (HP:0002017)** | Y |  | Y | Y |  |  |  |  |  |  |  |  |
| **hepatomegaly (HP:0002240)** | Y |  |  |  |  |  |  |  |  |  |  |  |
| **hypoglycemia (HP:0001943)** | Y | N | Y | Y | Y | Y | Y | Y | Y | Y | Y | Y |
| **lactic acidosis (HP:0003128)** |  |  | Y | Y | Y | Y | Y |  | Y | Y |  |  |
| **triggered by febrile illness (HP:0025215)** |  | Y |  | Y |  |  |  |  |  |  |  |  |
| **triggered by fasting (HP:0025212)** | Y |  |  |  |  |  |  |  |  |  |  |  |
| **others symptoms during decompensation** | sleepiness |  |  | lethargy |  | encephalopathy |  |  |  | encephalopathy |  | encephalopathy |
| **Other triggering factors** |  |  |  |  |  |  |  |  |  |  |  |  |
| **extrahepatic symptoms** | | | | | | | | | | | | |
| **cognitive impairment (HP:0100543)** | N | N | N | N |  | Y | N | N | Y | N | N | N |
| **muscular hypotonia (HP:0001252)** |  |  |  |  |  |  |  |  |  | Y | Y |  |
| **post-exertional symptom exacerbation (HP:0030973)** |  |  |  |  |  |  |  |  |  |  |  |  |
| **others** | failure to thrive |  | developmental difficulties |  |  | ataxia, ADD, delayed motor and language development, gastroesophageal reflux | spastic movements, seizures (either due to hypoglycemia or fever), difficulties reading and writing, difficulties with fine motor skills | ADHD, reading/writing difficulties, language development delay, difficulties in planning/executional abilities | seizures, gastroesophageal reflux, speech delay |  | decreased ability in fine motor skills, optic atrophy, suspected spinal fusion | high CK |
| **therapy** | | | | | | | | | | | | |
| **B1 (thiamine)** | N | N | N | N |  | N | Y | N | N | Y |  | N |
| **B2 (riboflavin)** | Y | Y | N | N |  | Y | Y | Y | Y | Y | Y | Y |
| **NAC** | N | N | N | N |  | N | N | N | N | N | N | N |
| **ketogenic diet** | N | N | N | N |  | N | N | N | N | N | N | N |
| **carnitine** | N | N | N | N |  | N | Y | N | N | Y | N | N |
| **lipoic acid** | N | N | N | N |  | N | N | N | N | N | N | N |
| **MCT** | N | N | N | N |  | N | N | N | N | N | N | N |
| **others** | N | N | N | N | SMOF, bicarbonate | atomoxetine, risperdal | N | amphetamine | levetiracetam | N | DCA | N |
| **improvement seen on:** |  |  |  |  |  |  |  |  |  |  |  |  |

DLD Dihydrolipoamide dehydrogenase, ID identification number, ELT Elevated liver transaminases, ALF acute liver failure, M male, F female, h hours, d days, m months, ys years, Y yes, N no, NAC N acetyl-cysteine, DCA dichloroacetate, MCT medium chain triglycerides, cMRI cranial magnetic resonance imaging, AD(H)D attention deficit (hyperactivity) disorder, CK creatinine kinase, US United States, UK United Kingdom

**Supplemental table S2:** Laboratory values for pH, lactate, AST, ALT, INR, albumin, bilirubin, GGT, ammonia and glucose during crises and in the interval for our cohort of individuals with DLD deficiency.

| **ID** | **Reference*** | **DLD-1** | | | | | | | | | | | | | | | | | |
| --- | --- | --- | --- | --- | --- | --- | --- | --- | --- | --- | --- | --- | --- | --- | --- | --- | --- | --- | --- |
| **age** |  | 4y5m | 4y9m | 4y10m | 6y2m | 6y5m | 6y10m | 6y11m | 7y | 7y2m | 7y4m | 7y4m | 7y6m | 7y6m | 7y7m | 7y8m | 7y8m | 7y9m | 7y10m |
| **during Crises (C)/ in the interval (I)** |  | C | C | C | C | C | C | C | C | C | C | C | C | I | C | C | C | C | C |
| **pH** | 7.35-7.45 | **7.48** | 7.35 | 7.37 | **7.30** | **7.27** | 7,35 | 7.37 | 7.38 | **7.27** | 7.35 | **7.32** | **7.29** |  | **7.32** | **7.28** | **7.33** | **7.31** | 7.36 |
| **lactate (plasma) (mmol/l)** | <1.70 | **7.33** | **4.11** | **3.33** | **5.55** | **5.99** | **3.99** | **4.55** | **2.55** | **4.55** | **5.00** | **4.88** | **5.00** |  | **4.88** | **3.89** | **6.22** | **4.44** | **6.44** |
| **AST (µkat/l)** | <0.65 |  |  | **1.43** |  | **33.81** | **2.98** | **1.12** | **1.01** | **1.03** | **5.77** | 0.45 | **0.82** | 0.30 | 0.60 | **1.03** | **0.70** | 0.42 | 0.48 |
| **ALT (µkat/l)** | <0.83 |  |  | **1.23** |  | **44.13** | **1.85** | 0.70 | 0.78 | **0.83** | **5.00** | 0.32 | 0.55 | 0.50 | 0.37 | 0.60 | **6.70** | 0.20 | 0.32 |
| **INR** | <1.20 |  |  |  |  | 1.17 | 1.16 |  |  | **1.44** | **1.45** | 1.08 | **1.28** |  | 1.15 | **1.28** |  |  |  |
| **albumin (g/l)** | 37.00-51.00 |  |  |  |  | 32.20 | 54.90 | 49.80 |  | 43.00 | 47.40 | **51.10** | 50.20 | 46.10 | 40.60 | 49.20 |  |  |  |
| **bilirubin (µmol/l)** | <17.10 |  |  | 10.26 |  | 13.68 | 11.97 | **17.10** |  | 6.84 | 6.84 | 8.55 | 8.55 | 6.84 | 5.13 |  |  |  |  |
| **GGT (µkat/l)** | <1.00 |  |  |  |  | **2.92** | 1.85 | 0.18 | 0.25 | 0.18 | 0.17 | 0.18 | 0.15 | 0.27 | 0.15 | 0.13 | 0.80 |  | 0.18 |
| **ammonia (µmol/l)** | <53.00 |  |  |  |  |  |  |  |  |  |  |  |  |  |  |  |  |  |  |
| **glucose (mmol/l)** | 3.90-5.60 | **12.3** | **8.1** | **7** |  | **3.3** | 4.8 |  |  | **2.6** | 4.83 | **3.0** | **2.94** |  | **3.11** | **2.11** | **2.77** | **2.0** | **3.22** |

| **ID** | **DLD-1** | | | | | | | | | | | | | | | | | |
| --- | --- | --- | --- | --- | --- | --- | --- | --- | --- | --- | --- | --- | --- | --- | --- | --- | --- | --- |
| **age** | 8y5m | 8y7m | 8y9m | 9y4m | 10y | 10y6m | 11y1m | 11y4m | 11y6m | 11y8m | 12y1m | 12y1m | 12y3m | 12y9m | 12y11m | 12y11m | 13y2m | 13y5m |
| **C/I** | I | C | C | C | C | I | C | C | I | C | I | C | C | C | I | C | C | C |
| **pH** | 7.36 | **7.32** | **7.34** | **7.23** | **7.35** | **7.34** | **7.27** | **7.31** | 7.38 | 7.38 | **7.34** | **7.35** | **7.40** | **7.34** | 7.35 | **7.33** | **7.35** | 7.38 |
| **lactate (plasma) (mmol/l)** | 1.22 | **3.00** | **7.66** | **7.66** | **4.22** | 0.78 | **10.66** | **5.44** | 0.89 | **1.89** | 1.00 | **3.11** | **1.89** | **3.89** | 0.67 | **2.66** | **2.44** | **1.78** |
| **AST (µkat/l)** | 0.4 | **0.85** | **2.08** | **1.72** | **10.04** | 0.30 | **12.14** | **2.02** | 0.33 | 0.42 | 0.32 | **2.32** | **0.77** | **25.46** | 0.28 | 0.58 | **3.1** | 0.62 |
| **ALT (µkat/l)** | 0.32 | 0.48 | **1.95** | **1.35** | **15.04** | 0.13 | **14.67** | **3.28** | 0.18 | 0.22 | 0.18 | **5.35** | **1.22** | **35.77** | 0.27 | 0.75 | **5.03** | **0.97** |
| **INR** |  |  | **1.62** | **1.49** | **1.33** | 1.02 | **1.73** | **1.52** | 1.02 | 1.13 | 0.97 | **1.24** | **1.23** | **1.56** | 0.98 | **1.49** | **1.31** | **1.21** |
| **albumin (g/l)** | 45.2 |  |  |  | **52.50** | 48.50 | **52.30** |  | 49.50 |  | 47.60 | 52.00 |  | 50.00 | 46.80 |  |  | 44.80 |
| **bilirubin (µmol/l)** | 6.84 |  |  |  | **22.23** | 6.84 | **27.36** | **18.80** | 5.13 |  |  | 11.97 | 11.97 |  | 5.99 | 14.36 | **20.18** | 13.17 |
| **GGT (µkat/l)** | 0.08 |  | 0.15 |  | 0.30 | 0.10 | 0.12 |  | 0.13 |  | 0.18 | 0.17 | 0.13 | 0.20 | 0.20 | 0.18 | 0.18 | 0.20 |
| **ammonia (µmol/l)** |  |  |  |  | <7.00 |  | 19.00 |  |  |  |  |  |  |  |  |  |  |  |
| **glucose (mmol/l)** | 5.44 | 5.16 | 5.66 | 3.94 | 3.94 | 4.77 | 5.66 | 4.16 | 5.16 | 4.77 | 3.50 | 3.50 | 5.22 | 4.11 | 5.22 | **2.50** | 4.05 | 5.11 |

| **ID** | **DLD-2** | | | | | | | | | | | **DLD-3** | | | | |
| --- | --- | --- | --- | --- | --- | --- | --- | --- | --- | --- | --- | --- | --- | --- | --- | --- |
| **age** | 12y | 16y | 17y | 19y | 20y | 20y | 20y | 21y | 21y | 21y | 24y | 1y6m | 3y | 8y11m | 9y1m | 11 y4m |
| **C/I** | C | C | I | C | C | C | C | C | C | C | I | C | C | C | C | I |
| **pH** |  |  |  |  |  |  |  |  |  |  |  |  |  |  |  |  |
| **lactate (plasma) (mmol/l)** | ↑ | **4.40** |  | **4.40** | **4.00** | **4.60** | **2.80** | **2.60** | ↑ | ↑ |  |  |  | 2.50 |  | 1.70 |
| **AST (µkat/l)** | N | **6.33** |  | **5.85** | **1.22** | **1.22** | N | ↑ | ↑ | **70.21** |  | **145.03** |  | **89.10** | **7.47** | 0.45 |
| **ALT (µkat/l)** | N | **19.85** |  | **7.83** | **2.97** | **2.97** | N | ↑ | ↑ | **80.02** |  | **133.36** |  | **58.38** | **29.56** | 0.28 |
| **INR** | N | **2.20** |  | N | N | N | N |  |  | N |  | N |  | 0.86 | 1.30 |  |
| **albumin (g/l)** |  |  |  |  |  |  |  |  |  |  |  |  |  | 43.00 |  |  |
| **bilirubin (µmol/l)** |  | **42.75** |  | **44.46** | **44.46** | **42.75** | **27.36** | ↑ | ↑ |  |  | N |  |  |  |  |
| **GGT (µkat/l)** |  |  |  |  |  |  |  |  |  |  |  |  |  | **0.95** | **3.83** | 0.28 |
| **ammonia (µmol/l)** |  | **252.00** |  |  |  |  |  |  |  |  |  | N |  | 17.00 | 15.00 |  |
| **glucose (mmol/l)** |  |  |  |  |  |  |  |  |  |  |  |  |  | **1.20** | **2.30** |  |

| **ID** | **DLD-4** | | | | | | | | | | | | **DLD-5** | | | | | |
| --- | --- | --- | --- | --- | --- | --- | --- | --- | --- | --- | --- | --- | --- | --- | --- | --- | --- | --- |
| **age** | 0m | 1m | 2,5m | 4m | 6,5m | 11,5m | 1y6m | 2y | 2y8m | 2y11m | 3y1m | 4y10m | 3 d | 3y2m | 3y9m | 5y9m | 6y1m | 6y5m |
| **C/I** | C | I | C | I | C | I | I | C | C | C | I | I | C | C | C | C | C | C |
| **pH** | **7.15** | 7.43 |  | 7.41 | 7.33 | 7.38 |  |  |  |  | 7,35 |  | **7.34** |  |  |  |  |  |
| **lactate (plasma) (mmol/l)** | **19.00** |  | **6.00** | **5.70** |  | **3.50** |  |  |  |  | **2.40** |  | **5,10** |  |  |  |  |  |
| **AST (µkat/l)** | **2.28** | **1.10** | **25.06** | **1.18** | **4.25** | **1.17** | **1.05** | **0.90** | **0.92** |  | **0.72** | 0.60 | **71.66** |  |  |  |  |  |
| **ALT (µkat/l)** | **1.35** | **0.97** | **17.67** | **1.05** | **2.57** | **0.98** | 0.70 | **0.97** | **3.70** |  | 0.35 | 0.23 | **75.40** |  |  |  |  |  |
| **INR** | **3.20** | **1.30** | **1.60** | 1.00 | **1.20** | **1.40** | 1.14 | **1.30** | 1.09 |  | 1.10 | 1.00 | **3.57** | **3.31** |  |  |  |  |
| **albumin (g/l)** | 43.20 | 38.60 | 40.60 | 39.70 | 38.10 | 40.10 | 49.00 | 40.70 | 43.00 |  | N | 47.00 |  |  |  |  |  |  |
| **bilirubin (µmol/l)** | **56.78** | 4.93 | 3.91 | 2.89 | 10.03 | 2.72 | 6.84 | 2.72 | 8.55 |  | N | 3.40 | **97.00** |  |  |  |  |  |
| **GGT (µkat/l)** | **0.98** | 0.53 | **0.92** | **0.70** | **4.23** | 0.27 |  | 0.27 |  |  | 0.22 | 0.20 | **1.43** |  |  |  |  |  |
| **ammonia (µmol/l)** | **102.70** | 24.00 | **71.70** | 16.00 |  |  |  |  |  |  |  |  | **333.00** | **100.00** |  |  |  |  |
| **glucose (mmol/l)** |  | 4.33 | 4.90 |  | **1.40** | **3.83** | 3.94 | 4.385 | 4.38 |  | 5.27 | 4.44 |  | **0.60** |  |  |  |  |

| **ID** | **DLD-6** | | | **DLD-7** | | | | | | | | | | | | | | | |
| --- | --- | --- | --- | --- | --- | --- | --- | --- | --- | --- | --- | --- | --- | --- | --- | --- | --- | --- | --- |
| **age** | 3y4m | 3y10m | 4y10m | 3d | 8d | 4m | 5m | 6m | 7m | 8m | 8m | 9m | 9m | 10 | 11m | 12m | 1y2m | 1y3m | 1y3m |
| **C/I** | C | C | C | I | C | C | C | C | C | C | I | C | C | C | I | C | I | C | C |
| **pH** |  |  |  |  | 7.46 | **7.30** | **7.42** |  |  | 7.37 | 7.41 |  | **7.31** | **7.24** | 7.38 | **7.32** | **7.28** | **7.27** | **7.21** |
| **lactate (plasma) (mmol/l)** | **12.80** |  |  |  | **8.20** | **10.00** | **3.80** | **5.30** | **4.40** | **8.00** | **3.80** | **6.90** | **5.50** | **9.10** | **6.50** | **5.40** | **6.30** | **8.70** | ↑ |
| **AST (µkat/l)** | **8.59** | **9.87** | **1.15** |  | **5.37** | **5.95** | **>50.00** | **0.68** | **0.63** |  | **0.70** | **0.63** | **0,75** | **0.72** |  | **0.82** | **0.68** | **0.87** | **45.01** |
| **ALT (µkat/l)** | **6.02** | **8.45** | **1.55** |  | **30.61** | **40.76** | **33.16** | **0.77** | **0.85** |  | **0.72** | 0.38 | 0.50 | 0.37 |  | 0.57 | 0.48 | **0.58** | **30.01** |
| **INR** | **1.78** | **1.69** | **1.74** |  | **>6.50** | **2.30** | **2.02** | **1.80** | **1.78** | 1.03 | 0.90 | 1.19 | **1.50** | 1.15 |  | **1.72** | 1.00 | **1.40** | **3.20** |
| **albumin (g/l)** | 43.00 |  |  |  | 27.00 | 44.00 | 39.00 |  | 36.00 |  | 43.00 | 37.00 | **34.00** |  |  |  | 44.00 |  | 41.00 |
| **bilirubin (µmol/l)** | 3.00 |  |  |  | **217.00** | 6.00 | 11.00 | 3.00 | 4.00 |  | 4.00 | 6.00 | 6.00 | 6.00 |  | 5.00 | 3.42 | 4.00 | 12.00 |
| **GGT (µkat/l)** | 0.18 |  |  |  | **5.00** | **0.78** | 0.57 | **0.83** | **0.87** |  | **0.72** | 0.50 | 0.47 | 0.37 |  |  | **0.58** | 0.50 | 0.45 |
| **ammonia (µmol/l)** | 36.00 |  |  |  | **203.00** | 46.00 | **71.00** | ↑ | **61.00** |  |  | **55.00** | 46.00 | **70.00** |  | 38.00 | **116.00** |  | 43.00 |
| **glucose (mmol/l)** |  |  |  |  | **2.70** | **<0.56** | 4.11 | **2.30** | 5.20 | 5.10 | 4.90 | 5.10 | 5.90 | **3.40** | **6.70** | **4.10** | 4.94 | 4.50 | 4.90 |

| **ID** | **DLD-7** | | | | | | | | | | | | | | | | | | |
| --- | --- | --- | --- | --- | --- | --- | --- | --- | --- | --- | --- | --- | --- | --- | --- | --- | --- | --- | --- |
| **age** | 1y8m | 1y9m | 1y10m | 1y11m | 2y | 2y2m | 2y6m | 2y7m | 2y9m | 2y10m | 3y1m | 3y4m | 3y9m | 4y7m | 4y9m | 4y10m | 4y10m | 4y11m | 5y |
| **C/I** | I | I | I | I | I | I | C | I | C | C | C | I | I | C | I | C | C | C | C |
| **pH** | **7.26** | **7.28** | **7.30** | **7.32** | **7.18** | **7.30** |  | **7.30** | **7.14** | **7.32** | **7.31** |  | 7.41 | 7.35 | 7.36 | 7.37 | 7.37 | **7.33** | 7.38 |
| **lactate (plasma) (mmol/l)** | **8.10** | **8.30** | **7.80** | **2.80** | **8.50** | **6.90** |  | **7.80** | **14.1** | **7.30** | **6.40** |  | **1.79** | **5.70** | **4.33** | **4.70** | **4.50** | **5.10** | **3.00** |
| **AST (µkat/l)** | **0.77** | **0.87** | **0.83** |  |  | **0.93** | ↑ | **0.77** | **3.98** | **1.88** | **20.52** | **0.87** | 0.58 | **0.68** | 0.70 | **1.35** | **0.70** | **0.73** | **0.80** |
| **ALT (µkat/l)** | **0.75** | **0.93** | **0.90** |  |  | **0.72** | ↑ | **0.60** | **6.27** | **3.02** | **41.84** | 0.40 | 0.30 | 0.42 | 0.42 | **0.73** | 0.40 | 0.37 | 0.43 |
| **INR** | 0.98 | 0.93 | 0.95 |  |  | 1.04 | **2.10** | 0.88 | **1.28** | **1.50** | 1.19 | 0.96 | 1.06 | 0.88 | 1.01 | 1.14 | 1.02 | 1.10 | 1.21 |
| **albumin (g/l)** |  | 48.00 |  |  |  | 50.00 | 36.00 | 52.00 | 47.00 | 42.00 | 45.00 | 51.00 | 45.80 |  | **51.30** |  | 40.00 | 51.00 | 39.00 |
| **bilirubin (µmol/l)** | <3.00 | 3.00 |  |  |  | 4.00 |  | 3.00 | 4.00 | 6.00 | 4.00 | 3.00 | 8.55 |  | 3.42 | 5.00 | 6.00 | 4.00 | 4.00 |
| **GGT (µkat/l)** | 0.28 | 0.28 | 0.27 |  |  | 0.25 |  | 0.28 | 0.23 | 0.27 | 0.50 | 0.20 | 0.22 | 0.23 | 0.30 | 0.15 | 0.17 | 0.22 | 0.18 |
| **ammonia (µmol/l)** | **90.00** | **77.00** |  | **84.00** |  | **92.00** | ↑ | **83.00** | **50.00** | **69.00** | **57.00** |  | 22.00 | **54.00** |  |  | **52.00** | **62.00** | **54.00** |
| **glucose (mmol/l)** | **6.30** | **6.20** | **6.10** | 5.80 |  | 5.00 |  | 5.90 | **4.10** |  | 5.50 | 5.10 | 5.38 | 6.20 | 5.61 | 5.90 | **6.20** | 5.80 | 5.00 |

| **ID** | **DLD-7** | | | | | | | | | | | | | | |
| --- | --- | --- | --- | --- | --- | --- | --- | --- | --- | --- | --- | --- | --- | --- | --- |
| **age** | 5y2m | 5y8m | 5y11m | 6y | 6y1m | 6y2m | 6y2m | 6y6m | 6y8m | 6y11m | 7 y | 7y | 7y1m | 7y2m | 7y4m |
| **C/I** | I | I | C | C | I | C | I | C | I | I | C | C | I | C | C |
| **pH** |  |  | 7.38 | 7.43 | 7.42 | 7.38 | 7.40 | 7.41 |  |  |  |  | 7.38 | 7.39 | **7.31** |
| **lactate (plasma) (mmol/l)** |  |  | **4.10** | 2.20 | **1.89** | **3.40** | 1.50 | **3.50** |  | 1.69 |  | **5.10** | **3.44** | **3.70** | 0.70 |
| **AST (µkat/l)** | **0.67** | **0.60** | **95.39** | **0.68** | 0.57 | **0.85** | **0.58** | **1.10** | **0.58** | **0.65** | **39.64** | **0.75** | 0.50 | **4.25** | **0.63** |
| **ALT (µkat/l)** | 0.32 | 0.33 | **89.45** | 0.48 | 0.35 | **0.95** | 0.45 | **1.13** | 0.35 | 0.40 | **41.37** | **3.03** | 0.52 | **4.63** | 0.40 |
| **INR** | 1.05 | 1.06 | **2.53** | 1.11 | 1.08 | **1.39** | 1.03 | **1.49** | 1.03 | 1.03 | **1.71** | 1.16 | 1.00 | 1.14 | **1.45** |
| **albumin (g/l)** | 52.00 | 49.00 | **32.00** | 43.00 | 50.80 | 42.00 | 48.00 |  | 46.00 | 50.00 | 38.00 | 41.00 | 49.00 | 38.00 | 42.00 |
| **bilirubin (µmol/l)** | 4.00 | 5.00 | 8.00 | 7.00 | 6.84 | 8.00 | 5.00 |  | 4.00 |  | 8.00 | 7.00 | 5.64 | 4.00 | 5.00 |
| **GGT (µkat/l)** | 0.22 | 0.23 | **1.35** | 0.27 | 0.28 | 0.22 | 0.22 | 0.17 | 0.20 | 0.22 | **0.75** | 0.50 | 0.33 | 0.23 | 0.22 |
| **ammonia (µmol/l)** |  |  | **53.00** | 32.00 |  | 39.00 |  |  |  | 41.00 | 36.00 |  | <7.00 | **72.00** |  |
| **glucose (mmol/l)** |  |  | **3.30** | 5.80 | 6.11 | **8.60** | 5.80 | 5.40 | 5.00 |  | **3.40** | 4.90 | 5.83 | 5.30 | **6.70** |

* reference values often age-and/or laboratory dependent, if values were above or underneath age- and laboratory-adapted reference value, they are marked fat

DLD Dihydrolipoamide dehydrogenase; ID identification number; N normal; ↑ elevated; ↓ reduced; y year(s); m month(s); d day(s); C crises; I interval; AST aspartate aminotransferase; ALT alanine aminotransferase, INR international normalized ratio; GGT gamma-glutamyl transferase

**Supplemental table S3:** Laboratory values for pH, lactate, AST, ALT, INR, albumin, bilirubin, GGT, ammonia (each maximum) and glucose (minimum) as reported in the literature (no laboratory values given in the publication by Pode-Shakked et al., 2024).

| **publication** | **ref.*** | **Elpeleg et al., 1995; Elpeleg et al., 1997; Shaag et al., 1999** | **Elpeleg et al., 1997; Shaag et al., 1999** | **Aptowitzer et al., 1997; Shaag et al., 1999** | **Shaag et al., 1999, Elpeleg et al., 1990** | | | | | **Shaag et al., 1999** | | | | **Shaag et al., 1999, Elpeleg et al., 1997** |
| --- | --- | --- | --- | --- | --- | --- | --- | --- | --- | --- | --- | --- | --- | --- |
| **ID** |  | Patient 2/ F1; II-3 | Patient 1/ F2; II-3 | F4; II-4 | F3; II-2 | F3; II-5 | F3; II-6 | F3; II-7 | F3; II-12 | F4; II-3 | F5; II-1 | F6; II-3 | F7; II-4 | F7; II-3 |
| **C/I** |  | C | C | C | C | C | C | C | C | C | C | C | C | C |
| **pH** | 7.35-7.45 | **7.15** | **↓** | **7.29** |  |  | **7.18** |  |  |  |  |  |  | **6.80** |
| **lactate (plasma) (mmol/l)** | <1.70 | **9.80** | **16.00** | **16.00** | ↑ | **26.00** | **12.20** | ↑ | ↑ | ↑ | ↑ | **9.70** | ↑ | **29.70** |
| **AST (µkat/l)** | <0.65 | **166.67** |  | **146.89** | **↑** | **↑** | **7.03** | **↑** | **↑** | **↑** | **↑** |  | **↑** | **9.85** |
| **ALT (µkat/l)** | <0.83 | **200.00** |  | **164.32** | **↑** | **↑** | **15.90** | **↑** | **↑** | **↑** | **↑** | **271.67** | **↑** | **27.34** |
| **INR** | <1.20 | **1.90** |  | **2.68** | **↑** | **↑** | **3.70** | **↑** | **↑** | **↑** | **↑** | **5.00** | **↑** | **↑** |
| **albumin (g/l)** | 37.00-51.00 |  |  | N |  |  |  |  |  |  |  |  |  |  |
| **bilirubin (µmol/l)** | <17.10 |  |  | N |  |  | **53.00** |  |  |  |  |  |  |  |
| **GGT (µkat/l)** | <10.00 |  |  | N |  |  |  |  |  |  |  |  |  |  |
| **ammonia (µmol/l)** | <53.00 |  | **↑** | N |  | **250.00** | **120.00** |  |  |  |  |  |  | **150.00** |
| **glucose (mmol/l)** | 3.90-5.60 |  |  |  | **↓** | **↓** | **2.8** | **↓** | **↓** |  |  |  |  |  |

| **publication** | **ref*** | **Hong et al., 2003** | | **Sansaricq et al., 2006** | **Brassier et al., 2013** | | | **Cameron et al., 2006** | **Hegarty et al., 2019** | **Neveu et al., 2020** | **Alfarsi et al., 2021** | | | |
| --- | --- | --- | --- | --- | --- | --- | --- | --- | --- | --- | --- | --- | --- | --- |
| **ID** |  | Patient 2 | Patient 4 |  | Patient 3 | Patient 4 | Patient 5 | Patient 1 | Patient 45 |  | Patient 1 | Patient 2 | Patient 3 | Patient 4 |
| **C/I** |  | C | C | C | C |  | C | C |  | C | C | C | C | C |
| **pH** | 7.35-7.45 | **↓** | **↓** | **7.32** |  |  | **7.10** | **7.18** |  | **7.17** |  |  |  |  |
| **lactate (plasma) (mmol/l)** | <1.70 | ↑ |  | **10.70** | **>10.00** |  | **6.00** | **12.50** |  | **11.80** | ↑ | ↑ | ↑ | N |
| **AST (µkat/l)** | <0.65 |  |  | **872.83** | **83.33** |  |  | **8.00** |  | **0.95** | **↑** | **↑** | **↑** | **↑** |
| **ALT (µkat/l)** | <0.83 |  |  | **694.67** | **83.33** |  |  | **8.93** |  | 0.52 | **↑** | **↑** | **↑** | **↑** |
| **INR** | <1.20 |  |  |  | **>6.00** |  |  |  |  | **1.20** |  |  |  |  |
| **albumin (g/l)** | 37.00-51.00 |  |  |  |  |  |  |  |  |  |  |  |  |  |
| **bilirubin (µmol/l)** | <17.10 |  |  |  | N |  |  |  |  |  |  |  |  |  |
| **GGT (µkat/l)** | <1.00 |  |  |  | N |  |  |  |  |  |  |  |  |  |
| **ammonia (µmol/l)** | <53.00 |  |  | 22.00 | **150.00** |  | **100.00** | **86.00** |  | 36.50 |  |  |  |  |
| **glucose (mmol/l)** | 3.90-5.60 |  |  | **1.30** | **2.70** |  | **2.10** | N |  | **2.44** | **↓** | N | N | N |

| **publication** | **ref*** | **Ramadža et al., 2021** | **Siri et al., 2022** | | **Wongkittichote et al., 2023** | | | **Moosavian et al., 2024** |
| --- | --- | --- | --- | --- | --- | --- | --- | --- |
| **ID** |  | Patient 1 | Subject 10 | Brother of Subject 10 | Patient 1 | Patient 3 | Patient 4 |  |
| **C/I** |  | C |  |  | C | C | C | C |
| **pH** | 7.35-7.45 | **7.12** |  |  |  |  |  |  |
| **lactate (plasma) (mmol/l)** | <1.70 | **19.70** |  | ↑ | ↑ | ↑ | ↑ | **3.89** |
| **AST (µkat/l)** | <0.65 | **297.49** | ↑ | ↑ |  |  |  | **20.83** |
| **ALT (µkat/l)** | <0.83 | **229.55** | ↑ | ↑ |  |  |  | **27.33** |
| **INR** | <1.20 | **3.50** |  |  |  |  |  | 1.00 |
| **albumin (g/l)** | 37.00-51.00 |  |  |  |  |  |  |  |
| **bilirubin (µmol/l)** | <17.10 | **71.00** |  |  |  |  |  | **34.00** |
| **GGT (µkat/l)** | <1.00 | **0.57** |  |  |  |  |  |  |
| **ammonia (µmol/l)** | <53.00 | **387.7** |  | ↑ |  |  |  | **70.50** |
| **glucose (mmol/l)** | 3.90-5.60 | **7.40** |  |  |  | **↓** |  |  |

* reference values often age-and/or laboratory dependent, if values were above or underneath age- and laboratory-adapted reference value, they are marked fat

Ref reference; ID identification number; N normal; ↑ elevated; ↓ reduced; y year(s); m month(s); d day(s); C crises; I interval; AST aspartate aminotransferase; ALT alanine aminotransferase, INR international normalized ratio; GGT gamma-glutamyl transferase

**Supplemental Table S4:** Laboratory values of selected amino acids (plasma) and selected organic acids (urine) during crises and during the interval for our cohort of individuals with DLD deficiency.

| **ID** | **ref.*** | **DLD-1** | | | | | | | | | | | | | | | | |
| --- | --- | --- | --- | --- | --- | --- | --- | --- | --- | --- | --- | --- | --- | --- | --- | --- | --- | --- |
| **age** |  | 6y5m | 7y4m | 7y6m | 7y8m | 8y5m | 8y7m | 8y9m | 9y4m | 10y | 10y6m | 11y1m | 11y6m | 11y8m | 12y1m | 12y1m | 12y3m | 12y11m |
| **C/I** |  | C | C | I | C | I | C | C | C | C | I | C | I | C | I | C | C | I |
| **lactate/creatinine ratio (urine) (mmol/mol creatinine)** | 0-431 | 15 | 15 | 21 |  | 10 | 152 | 12 | **647** |  | 25 | 44 | 4 | 2 | 120 | 27 | 24 | 17 |
| **selected amino acids (plasma) (µmol/l)** | | | | | | | | | | | | | | | | | | |
| alanine | 130-547 | 409 | 347 | 216 | 529 | 268 | 207 | **618** | **865** | 494 | 229 | **1256** | 256 | 186 | 357 | 333 |  | 246 |
| glutamate | 10-150 | 63 | 130 | 48 | **323** | 27 | 60 | **979** | **2106** | **363** | 43 | **3205** | 36 | **153** | 59 | **153** |  | 54 |
| glutamine | 254-823 | 581 | **1839** | 562 | **1170** | 595 | **1173** | **2311** | **1650** | **1209** | 729 | **2449** | 683 | **849** | 624 | **1220** |  | **885** |
| citrulline | 1-46 | 15 | **169** | 28 | **111** | 34 | **150** | **268** | **311** | **222** | **47** | **352** | **48** | **110** | 29 | **177** |  | 45 |
| leucine | 49-216 | 55 | 112 | 77 | **397** | 63 | **418** | **430** | **379** | **352** | 119 | **512** | 92 | 193 | 90 | **432** |  | 104 |
| isoleucine | 22-107 | 39 | 43 | 28 | **175** | 40 | **207** | **188** | **175** | **173** | 52 | **230** | 42 | 95 | 33 | **221** |  | 38 |
| valine | 80-321 | 107 | 237 | 115 | **487** | 130 | **542** | **547** | **507** | **476** | 203 | **635** | 153 | 280 | 170 | **540** |  | 170 |
| lysine | 50-284 | 88 | 193 | 72 | 68 | 99 | 59 | 73 | 79 | 61 | 100 | 120 | 84 | **35** | 123 | 63 |  | 85 |
| proline | 60-340 | 147 | 250 | 191 | **392** | 154 | 125 | 294 | **441** | 222 | 141 | **490** | 130 | 168 | 178 | 242 |  | 196 |
| glycine | 110-343 | **477** | **532** | 318 | 162 | **404** | 150 | 292 | 281 | 167 | 338 | **391** | **453** | 167 | **375** | 145 |  | **425** |
| allo-isoleucine | <1 | <1 | <1 | <1 | **13,5** | <1 | **7** | <1 | <1 | <1 | <1 | <1 | <1 | <1 | <1 | **7** |  | <1 |
| **selected organic acids (urine) (mmol/mol creatinine)** | | | | | | | | | | | | | | | | | | |
| ketonic acid (µmol/l) |  | 204 |  | 88 | **1418** |  |  |  |  |  |  |  |  |  |  |  |  |  |
| fumaric acid | 0-21 | 4 |  | 1 |  | 0 | 5 | 4 | 16 |  | 1 | 3 | 0 | 0 | 1 | 1 | 1 | 0 |
| succinic acid | 0-142 | 17 |  | 11 |  | 6 | 34 | 41 | 21 |  | 12 | 15 | 5 | 7 | 3 | 6 | 8 | 4 |
| 2-oxo-glutaric acid/ 2-ketoglutaric acid | 0-514 | 86 |  | 41 |  | 13 | 1 | 42 | 317 |  | 5 | 20 | 1 | 5 | 2 | 1 | 7 | 2 |
| 5-oxoproline acid | 0-80 | 53 |  | 80 |  | 48 | **115** | 33 | 45 |  | 26 | 34 | 11 | 8 | 17 | 30 | 39 | 23 |
| 2-hydroxy-3-methylvaleric acid | 0-2 | 0 |  | 0 |  | 0 | 1 | 0 | 0 |  | 0 | 0 | 0 | 0 | 0 | 0 | 0 | 0 |
| 2-ketoisocaproic acid | 0-3 | 1 |  | 1 |  | 0 | **9** | 1 | **11** |  | 0 | 3 | 0 | 0 | 0 | 1 | 0 | 0 |
| 2-hydroxyisovaleric acid/ 2-hydroxy-3-methylbutyric acid | 0-7 | **21** |  | 1 |  | 1 | **30** | 7 | **9** |  | 0 | **8** | 0 | 0 | 0 | 3 | 2 | 0 |
| 2-oxo-adipic acid/ 2-ketoadipic acid | 0-25 |  |  |  |  | 0 | 24 | 1 | **114** |  | 0 | 17 | 0 | 1 | 0 | 0 | 1 | 0 |
| 2-hydroxy-adipic acid | 0-20 |  |  |  |  | 0 | **103** | 1 | **118** |  | 1 | **167** | 0 | 2 | 0 | **30** | 6 | 0 |

| **ID** | **ref.*** | **DLD-2** | | | | | **DLD-3** | | **DLD-4** | | | | | | | |
| --- | --- | --- | --- | --- | --- | --- | --- | --- | --- | --- | --- | --- | --- | --- | --- | --- |
| **age** |  | 17 | 19 | 20 | 21 | 24 | 8y11m | 11 y4m | 0 m | 1 m | 2 m | 4 m | 6 m | 11 m | 2 y | 4y10m |
| **C/I** |  | I | C | C | C | I | C | I | C | I | C | I | C | I | C | I |
| **lactate/creatinine ratio (urine) (mmol/mol creatinine)** | 0-431 |  |  |  |  |  |  |  | **1544** |  |  |  |  |  |  |  |
| **selected amino acids (plasma) (µmol/l)** | | | | | | | | | | | | | | | | |
| alanine | 130-547 |  |  |  |  |  | 354 | 361 | **1264** | **571** |  | **581** |  | 397 | 253 | 386 |
| glutamate | 10-150 |  |  |  |  |  |  |  | **264** | **498** |  | **384** |  | **464** | 81 | 114 |
| glutamine | 254-823 |  | N |  |  |  | 119 | 123 | **3863** | 417 |  | **1074** |  | **946** | 544 | 579 |
| citrulline | 1-46 |  | N |  |  |  | 35 | 45 | 42 | 33 |  | **57** |  | **78** | 22 | 31 |
| leucine | 49-216 |  | N |  |  |  | 98 | 140 | **241** | **299** |  | 184 |  | **260** | 137 | 96 |
| isoleucine | 22-107 |  | N |  |  |  | 77 | 90 | 86 | **143** |  | 93 |  | **118** | 58 | 42 |
| valine | 80-321 |  | N |  | 261 |  | **107** | 232 | 315 | **341** |  | 208 |  | **352** | 210 | 156 |
| lysine | 50-284 |  |  |  |  |  |  |  | **311** | 163 |  | 110 |  | 63 | 105 | 57 |
| proline | 60-340 |  |  |  |  |  |  |  | **1428** | **466** |  | 297 |  | 262 | 212 | 173 |
| glycine | 110-343 |  |  |  |  |  | 370 | 307 | **641** | **350** |  | 302 |  | 325 | 205 | 314 |
| allo-isoleucine | <1 |  |  |  |  |  |  |  |  |  |  |  |  |  |  |  |
| others |  |  |  |  |  |  | tyrosine 37 (↓) |  |  |  |  |  |  |  |  |  |
| **selected organic acids (urine) (mmol/mol creatinine)** | | | | | | | | | | | | | | | | |
| ketonic acid (µmol/l) |  | N |  |  |  | N | N | N |  |  |  |  |  |  |  |  |
| fumaric acid | 0-21 | N |  |  |  | N | N | N |  |  | N |  | 10 |  | N |  |
| succinic acid | 0-142 | N |  |  |  | N | N | N |  |  | N |  | **666** |  | N |  |
| 2-oxo-glutaric acid/ 2-ketoglutaric acid | 0-514 | N |  |  |  | N | N | N | **1089** |  | N | **1964** | 24 |  | N |  |
| 5-oxoproline acid | 0-80 | N |  | 44 |  | N | N | N |  |  | N |  |  |  | N |  |
| 2-hydroxy-3-methylvaleric acid | 0-2 | N |  |  |  | N | N | N |  |  | N |  |  |  | N |  |
| 2-ketoisocaproic acid | 0-3 | N |  |  |  | N | N | N |  |  | N |  |  |  | N |  |
| 2-hydroxyisovaleric acid/ 2-hydroxy-3-methylbutyric acid | 0-7 | N |  |  |  | N | N | N |  |  | N |  |  |  | N |  |
| 2-oxo-adipic acid/ 2-ketoadipic acid | 0-25 | N | 12 | **43** |  | N | N | N |  |  | N |  |  |  | N |  |
| 2-hydroxy-adipic acid | 0-20 | N | **50** | **107** |  | N | N | N |  |  | N |  |  |  | N |  |
| others |  |  |  |  |  |  |  |  |  |  |  |  | Isocitrate 3 |  |  |  |

| **ID** | **ref.*** | **DLD-5** | | | | | | **DLD-6** | | | **DLD-7** | | | | | | | |  |
| --- | --- | --- | --- | --- | --- | --- | --- | --- | --- | --- | --- | --- | --- | --- | --- | --- | --- | --- | --- |
| **age** |  | 3d | 3y2m | 3y9m | 5y9m | 6y1m | 6y5m | 3y4m | 3y10m | 4y10m | 3d | 8d | 5m | 1y3m | 1y5m | 1y8m | 1y9m | 1y11m |  |
| **C/I** |  | C | C | C | C | C | C | C | C | C | I | C | C | C | I | I | I | I |  |
| **lactate/creatinine ratio (urine) (mmol/mol creatinine)** | 0-431 | 263 |  |  |  |  |  | **2390** | **6130** | **4070** |  |  |  |  |  |  |  |  |  |
| **selected amino acids (plasma) (µmol/l)** | | | | | | | | | | | | | | | | | | | |
| alanine | 130-547 |  |  |  |  |  |  |  | **853** | 538 |  | 296 | 334 | 248 |  | 353 | 360 | 279 |  |
| glutamate | 10-150 |  |  |  |  |  |  |  | **541** | **241** |  | 79 | 62 | 99 |  | 82 | 74 | 46 |  |
| glutamine | 254-823 | **2975** |  |  |  |  |  |  | 708 | 691 |  | 450 | 744 | 466 | ↑ | **927** | **985** | 777 |  |
| citrulline | 1-46 | **466** | **352** | **330** | **281** | **142** | **144** |  | **108** | **188** |  | 23 | 20 | 28 |  | 28 | 34 | 28 |  |
| leucine | 49-216 | 182 | **352** | **490** | **272** | **251** | **322** |  | **442** | **235** | **458** | **37** | 88 | 112 |  | 170 | 195 | 122 |  |
| isoleucine | 22-107 |  |  |  |  |  |  |  | **220** | 101 | **458** | 78 | 54 | 47 |  | 74 | 92 | 54 |  |
| valine | 80-321 |  |  |  |  |  |  |  | **381** | 285 | 183 | 133 | 126 | 175 |  | 289 | **315** | 208 |  |
| lysine | 50-284 |  |  |  |  |  |  |  | 153 | **45** |  | 134 | 163 | **31** |  | 98 | 111 | 70 |  |
| proline | 60-340 |  |  |  |  |  |  |  | 211 | 200 |  | 120 | 163 | 120 |  | 140 | 148 | 119 |  |
| glycine | 110-343 |  |  |  |  |  |  |  | 280 | 158 |  | 220 | 334 | 145 |  | **474** | **483** | 329 |  |
| allo-isoleucine | <1 |  |  |  |  |  |  |  | **8,4** | **3,7** |  | <1 |  | <1 |  | **4** | <1 | <1 |  |
| **selected organic acids (urine) (mmol/mol creatinine)** | | | | | | | | | | | | | | | | | | | |
| ketonic acid (µmol/l) |  |  |  |  |  |  |  |  |  |  |  |  |  |  |  |  |  |  |  |
| fumaric acid | 0-21 |  |  |  |  |  |  |  |  |  |  |  |  |  |  |  |  |  |  |
| succinic acid | 0-142 |  |  |  |  |  |  |  |  |  |  |  |  |  |  |  |  |  |  |
| 2-oxo-glutaric acid/ 2-ketoglutaric acid | 0-514 | **743** |  |  |  |  |  |  |  |  |  |  |  |  |  |  |  |  |  |
| 5-oxoproline acid | 0-80 |  |  |  |  |  |  |  |  |  |  |  |  |  |  |  |  |  |  |
| 2-hydroxy-3-methylvaleric acid | 0-2 |  |  |  |  |  |  |  |  |  |  |  |  |  |  |  |  |  |  |
| 2-ketoisocaproic acid | 0-3 |  |  |  |  |  |  |  |  |  |  |  |  |  |  |  |  |  |  |
| 2-hydroxyisovaleric acid/ 2-hydroxy-3-methylbutyric acid | 0-7 |  |  |  |  |  |  | **17** | **69** | **73** |  |  |  |  |  |  |  |  |  |
| 2-oxo-adipic acid/ 2-ketoadipic acid | 0-25 |  |  |  |  |  |  | **67** |  |  |  |  |  |  |  |  |  |  |  |
| 2-hydroxy-adipic acid | 0-20 | **32** |  |  |  |  |  |  |  |  |  |  |  |  |  |  |  |  |  |

| **ID** | **ref.*** | **DLD-7** | | | | | | | | |
| --- | --- | --- | --- | --- | --- | --- | --- | --- | --- | --- |
| **age** |  | 2y9m | 2y10m | 3y1m | 3y9m | 4y9m | 4y10m | 6y1m | 6y11m | 7y1m |
| **C/I** |  | C | C | C | I | I | C | I | I | I |
| **lactate/creatinine ratio (urine) (mmol/mol creatinine)** | 0-431 |  |  |  | 16 | 19 |  | 71 |  | 69 |
| **selected amino acids (plasma) (µmol/l)** | | | | | | | | | | |
| alanine | 130-547 | 368 | 272 | 378 | 492 | 363 | 325 | 521 | N | 466 |
| glutamate | 10-150 | 38 | 28 | 40 | 46 | 36 | 54 | 38 | N | 46 |
| glutamine | 254-823 | 457 | 448 | 595 | 651 | 782 | 685 | 620 | N | 636 |
| citrulline | 1-46 | 38 | 7 | 31 | 36 | 43 | 37 | 25 | N | 31 |
| leucine | 49-216 | 97 | 67 | 162 | 161 | 105 | 87 | 97 | N | 102 |
| isoleucine | 22-107 | 37 | 25 | 92 | **108** | 67 | 41 | 54 | N | 47 |
| valine | 80-321 | 164 | 85 | 229 | 221 | 181 | 136 | 136 | N | 158 |
| lysine | 50-284 | 77 | 61 | 167 | 169 | 92 | 87 | 129 | N | 98 |
| proline | 60-340 | 115 | 92 | 142 | 188 | 158 | 125 | 129 | N | 171 |
| glycine | 110-343 | **363** | 234 | **411** | 339 | 340 | 346 | **421** | N | **398** |
| allo-isoleucine | <1 | <1 | <1 | <1 | <1 | <1 | <1 | <1 | N | <1 |
| **selected organic acids (urine) mmol/mol creatinine)** | | | | | | | | | | |
| ketonic acid (µmol/l) |  |  |  |  | 51 | 33 |  |  | N | 21 |
| fumaric acid | 0-21 |  |  |  | 2 | 1 |  | 1 | N | 0 |
| succinic acid | 0-142 |  |  |  | 78 | 20 |  | 47 | N | 73 |
| 2-oxo-glutaric acid/ 2-ketoglutaric acid | 0-514 |  |  |  | 25 | 33 |  | 9 | N | 2 |
| 5-oxoproline acid | 0-80 |  |  |  | 32 | 29 |  | 26 | N | 14 |
| 2-hydroxy-3-methylvaleric acid | 0-2 |  |  |  | 0 | 0 |  | 0 | N | 0 |
| 2-ketoisocaproic acid | 0-3 |  |  |  | 1 | 0 |  | 0 | N | 0 |
| 2-hydroxyisovaleric acid/ 2-hydroxy-3-methylbutyric acid | 0-7 |  |  |  | 0 | 0 |  | 0 | N | 0 |
| 2-oxo-adipic acid/ 2-ketoadipic acid | 0-25 |  |  |  | 1 | 1 |  | 0 | N | 0 |
| 2-hydroxy-adipic acid | 0-20 |  |  |  | 2 | 1 |  | 0 | N | 0 |

* reference values often age-and/or laboratory dependent, if values were above or underneath age- and laboratory-adapted reference value, they are marked fat

Ref reference; ID identification number; N normal; ↑ elevated; ↓ reduced; y year(s); m month(s); d day(s); C crises; I interval.

**Supplemental Table S5:** Laboratory values of selected amino acids (plasma) and selected organic acids (urine) during crises and during the interval in all previously reported individuals with DLD deficiency (no laboratory values given in the publication by Pode-Shakked et al., 2024).

| **publication** | **ref.*** | **Elpeleg et al., 1995; Elpeleg et al., 1997; Shaag et al., 1999** | **Elpeleg et al., 1997; Shaag et al., 1999** | **Aptowitzer et al., 1997; Shaag et al., 1999** | **Shaag et al., 1999, Elpeleg et al., 1990** | | | | | **Shaag et al., 1999** | | | | **Shaag et al., 1999, Elpeleg et al., 1997** |
| --- | --- | --- | --- | --- | --- | --- | --- | --- | --- | --- | --- | --- | --- | --- |
| **ID** |  | Patient 2/ F1; II-3 | Patient 1/ F2; II-3 | F4; II-4 | F3; II-2 | F3; II-5 | F3; II-6 | F3; II-7 | F3; II-12 | F4; II-3 | F5; II-1 | F6; II-3 | F7; II-4 | F7; II-3 |
| **C/I** |  | C | | | C, normal in I | | | | | C | | | | |
| **lactate/creatinine ratio (urine) (mmol/mol creatinine)** | 0-431 |  |  | ↑ |  |  |  |  |  |  |  |  |  | ↑ |
| **selected amino acids (plasma) (µmol/l)** | | | | | | | | | | | | | | |
| alanine | 130-547 | ↑ |  | **677** |  | 269 | **829** |  |  |  |  |  |  |  |
| glutamate | 10-150 |  |  | N |  |  |  |  |  |  |  |  |  |  |
| glutamine | 254-823 |  |  | N |  | **1202** | **1024** |  |  |  |  |  |  |  |
| citrulline | 1-46 | N |  | N |  | **221** | **304** |  |  |  |  |  |  |  |
| leucine | 49-216 | N | ↑* | N | ↑* | **188** | **389** | ↑* | | | | | | **1.5 to 2.5 times upper normal range** |
| isoleucine | 22-107 | N |  | N |  | **88** | **208** |  |  |  |  |  |  |  |
| valine | 80-321 | N |  | N |  | 232 | **557** |  |  |  |  |  |  |  |
| lysine | 50-284 |  |  | N |  | 63 | 140 |  |  |  |  |  |  |  |
| proline | 60-340 |  |  | N |  |  |  |  |  |  |  |  |  |  |
| glycine | 110-343 |  |  | N |  |  |  |  |  |  |  |  |  |  |
| allo-isoleucine | <1 |  |  | N |  |  |  |  |  |  |  |  |  |  |
| **selected organic acids (urine) (mmol/mol creatinine)** | | | | | | | | | | | | | | |
| ketonic acid (µmol/l) |  | **6610** |  |  |  |  |  |  |  |  |  |  |  |  |
| fumaric acid | 0-21 |  |  | ↑ |  |  |  |  |  |  |  |  |  |  |
| succinic acid | 0-142 |  |  |  |  |  |  |  |  |  |  |  |  |  |
| 2-oxo-glutaric acid | 0-514 | **4.14** | ↑* | ↑ | ↑* | | | | | | | | | |
| 5-oxoproline acid | 0-80 |  |  |  |  |  |  |  |  |  |  |  |  |  |
| 2-hydroxy-3-methylvaleric acid | 0-2 |  |  |  |  |  |  |  |  |  |  |  |  |  |
| 2-ketoisocaproic acid | 0-3 |  |  |  |  |  |  |  |  |  |  |  |  |  |
| 2-hydroxyisovaleric acid | 0-7 |  |  |  |  |  |  |  |  |  |  |  |  |  |
| 2-oxo-adipic acid | 0-25 |  |  |  |  | **85** | **56** |  |  |  |  |  |  |  |
| 2-hydroxy-adipic acid | 0-20 |  |  |  |  | **74** | **89** |  |  |  |  |  |  |  |

| **publication** | **ref.*** | **Hong et al., 2003** | | **Sansaricq et al., 2006** | **Brassier et al., 2013** | | | **Cameron et al., 2006** | **Hegarty et al., 2019** | **Neveu et al., 2020** | **Alfarsi et al., 2021** | |
| --- | --- | --- | --- | --- | --- | --- | --- | --- | --- | --- | --- | --- |
| **ID** |  | Patient 2 | Patient 4 |  | Patient 3 | Patient 4 | Patient 5 | Patient 1 | Patient 45 |  | Patient 1 | Patient 2 |
| **C/I** |  | C | C | C (normal in interval) | C |  | C | C |  | C | C | C |
| **lactate/creatinine ratio (urine) (mmol/mol creatinine)** | 0-431 |  |  | ↑ |  |  | **14** | ↑ |  | ↑ |  |  |
| **selected amino acids (plasma) (µmol/l)** | | | | | | | | | | | | |
| alanine | 130-547 |  |  |  | ↑ |  | **691** | **1136** |  | **881** |  |  |
| glutamate | 10-150 |  |  |  | N |  |  |  |  | **452** |  |  |
| glutamine | 254-823 |  |  | **1693** | N |  |  |  |  | **1180** |  |  |
| citrulline | 1-46 |  |  | **↑** | N |  |  |  |  | **179** |  |  |
| leucine | 49-216 |  |  | **285** | N |  |  | ↑ |  | **338** | N | N |
| isoleucine | 22-107 |  |  | **137** | N |  |  | ↑ |  | **157** | N | N |
| valine | 80-321 |  |  | **374** | N |  |  | ↑ |  | **498** | N | N |
| lysine | 50-284 |  |  |  | N |  |  |  |  |  |  |  |
| proline | 60-340 |  |  |  | N |  |  |  |  | **363** |  |  |
| glycine | 110-343 |  |  |  | N |  |  |  |  |  |  |  |
| allo-isoleucine | <1 |  |  | N | N |  |  |  |  | **10** |  |  |
| others |  |  |  |  |  |  |  | elevation of all amino acids |  |  |  |  |
| **selected organic acids (urine) (mmol/mol creatinine)** | | | | | | | | | | | | |
| ketonic acid (µmol/l) |  |  |  | ↑ | N |  |  |  |  | 4 + | ↑ |  |
| fumaric acid | 0-21 |  |  |  | N |  |  |  |  |  |  |  |
| succinic acid | 0-142 |  |  |  | N |  |  |  |  |  |  |  |
| 2-oxo-glutaric acid | 0-514 |  |  | ↑ | N |  |  | ↑ |  | ↑ |  |  |
| 5-oxoproline acid | 0-80 |  |  |  | N |  |  |  |  |  |  |  |
| 2-hydroxy-3-methylvaleric acid | 0-2 |  |  |  | N |  |  |  |  | ↑ |  |  |
| 2-ketoisocaproic acid | 0-3 |  |  |  | N |  |  |  |  |  |  |  |
| 2-hydroxyisovaleric acid | 0-7 |  |  |  | N |  |  |  |  | ↑ |  |  |
| 2-oxo-adipic acid | 0-25 |  |  |  | N |  |  |  |  | ↑ |  |  |
| 2-hydroxy-adipic acid | 0-20 | ↑ |  |  | N |  |  |  |  | ↑ |  |  |

| **publication** | **ref.*** | **Alfarsi et al., 2021** | | **Ramadža et al., 2021** | **Siri et al., 2022** | | **Wongkittichote et al., 2023** | | | **Moosavian et al., 2024** |
| --- | --- | --- | --- | --- | --- | --- | --- | --- | --- | --- |
| **ID** |  | Patient 3 | Patient 4 | Patient 1 | Subject 10 | Brother of Subject 10 | Patient 1 | Patient 3 | Patient 4 |  |
| **C/I** |  | C | C | C | C/I | C | C | C | C | C |
| **lactate/creatinine ratio (urine) (mmol/mol creatinine)** | 0-431 |  |  |  |  |  | ↑ | ↑ | ↑ |  |
| **selected amino acids (plasma) (µmol/l)** | | | | | | | | | | |
| alanine | 130-547 |  |  |  | 387 |  | ↑ | N | N |  |
| glutamate | 10-150 |  |  |  |  |  | ↑ | ↑ | N |  |
| glutamine | 254-823 |  |  |  |  | **1880** | ↑ | ↑ | ↑ |  |
| citrulline | 1-46 |  |  |  | **27** | **554** | N | ↑ | ↑ | N |
| leucine | 49-216 | N | ↑ |  | 213 | **572** | N | ↑ | N | N |
| isoleucine | 22-107 | N | ↑ |  |  | **267** | ↑ | N | N | N |
| valine | 80-321 | N | ↑ |  | **256** | **538** | N | N | N | N |
| lysine | 50-284 |  |  |  |  |  | N | **↓** | ↑ |  |
| proline | 60-340 |  |  |  |  |  | N | N | N |  |
| glycine | 110-343 |  |  |  |  |  | N | ↑ | N |  |
| allo-isoleucine | <1 |  |  |  | **2,8** | **28** | N |  | N | ↑ |
| **selected organic acids (urine) (mmol/mol creatinine)** | | | | | | | | | | |
| ketonic acid (µmol/l) |  |  |  |  |  |  |  |  |  |  |
| fumaric acid | 0-21 |  |  |  |  |  |  | ↑ | ↑ |  |
| succinic acid | 0-142 |  |  |  |  |  |  | ↑ | ↑ |  |
| 2-oxo-glutaric acid | 0-514 |  |  |  |  |  | ↑ | ↑ | ↑ | ↑ |
| 5-oxoproline acid | 0-80 |  |  |  |  |  |  |  |  |  |
| 2-hydroxy-3-methylvaleric acid | 0-2 |  |  |  |  |  |  |  |  |  |
| 2-ketoisocaproic acid | 0-3 |  |  |  |  |  |  |  |  |  |
| 2-hydroxyisovaleric acid | 0-7 |  |  |  |  | **>1000** |  |  |  |  |
| 2-oxo-adipic acid | 0-25 |  |  |  |  |  |  |  | ↑ | ↑ |
| 2-hydroxy-adipic acid | 0-20 |  |  |  |  |  |  |  | ↑ | ↑ |

* reference values often age-and/or laboratory dependent, if values were above or underneath age- and laboratory-adapted reference value, they are marked fat

Ref reference; ID identification number; N normal; ↑ elevated; ↓ reduced; y year(s); m month(s); d day(s); C crises; I interval; ↑* infrequent elevation in the entire cohort
